# Organelle-targeted biosensors reveal distinct oxidative events during pattern-triggered immune responses

**DOI:** 10.1093/plphys/kiac603

**Published:** 2022-12-30

**Authors:** Dominique Arnaud, Michael J Deeks, Nicholas Smirnoff

**Affiliations:** Biosciences, College of Life and Environmental Sciences, University of Exeter, Exeter EX4 4QD, UK; Biosciences, College of Life and Environmental Sciences, University of Exeter, Exeter EX4 4QD, UK; Biosciences, College of Life and Environmental Sciences, University of Exeter, Exeter EX4 4QD, UK

## Abstract

Reactive oxygen species are produced in response to pathogens and pathogen-associated molecular patterns, as exemplified by the rapid extracellular oxidative burst dependent on the NADPH oxidase isoform RESPIRATORY BURST OXIDASE HOMOLOG D (RBOHD) in Arabidopsis (*Arabidopsis thaliana*). We used the H_2_O_2_ biosensor roGFP2-Orp1 and the glutathione redox state biosensor GRX1-roGFP2 targeted to various organelles to reveal unsuspected oxidative events during the pattern-triggered immune response to flagellin (flg22) and after inoculation with *Pseudomonas syringae*. roGFP2-Orp1 was oxidized in a biphasic manner 1 and 6 h after treatment, with a more intense and faster response in the cytosol compared to chloroplasts, mitochondria, and peroxisomes. Peroxisomal and cytosolic GRX1-roGFP2 were also oxidized in a biphasic manner. Interestingly, our results suggested that bacterial effectors partially suppress the second phase of roGFP2-Orp1 oxidation in the cytosol. Pharmacological and genetic analyses indicated that the pathogen-associated molecular pattern-induced cytosolic oxidation required the BRI1-ASSOCIATED RECEPTOR KINASE (BAK1) and BOTRYTIS-INDUCED KINASE 1 (BIK1) signaling components involved in the immune response but was largely independent of NADPH oxidases RBOHD and RESPIRATORY BURST OXIDASE HOMOLOG F (RBOHF) and apoplastic peroxidases peroxidase 33 (PRX33) and peroxidase 34 (PRX34). The initial apoplastic oxidative burst measured with luminol was followed by a second oxidation burst, both of which preceded the two waves of cytosolic oxidation. In contrast to the cytosolic oxidation, these bursts were RBOHD-dependent. Our results reveal complex oxidative sources and dynamics during the pattern-triggered immune response, including that cytosolic oxidation is largely independent of the preceding extracellular oxidation events.

## Introduction

Reactive oxygen species (ROS) such as hydrogen peroxide (H_2_O_2_) are signaling molecules involved in various biological processes such as development and responses to environmental stresses. The production of ROS in response to pathogens and pathogen-associated molecular patterns (PAMPs) is common across many groups of organisms. In plants, an early PAMP-triggered immunity (PTI) response within minutes is a transient apoplastic oxidative burst mediated by plasma membrane nicotinamide adenine dinucleotide phosphate (NADPH) oxidases (termed respiratory burst oxidase homolog [RBOH] in plants) and cell wall peroxidases (PRXs) (Torres et al., 2002; Bindschedler et al., 2006; Zhang et al., 2007; Daudi et al., 2012). Extracellularly produced H_2_O_2_ diffuses into the cell probably through aquaporin to activate downstream defense responses such as stomatal closure or callose deposition (Tian et al., 2016; Rodrigues et al., 2017). The perception of PAMPs by plasma membrane receptor kinases (RKs) activates the co-receptor BRI1-associated receptor kinase (BAK1) and the cytosolic kinase BIK1 which in turn phosphorylates and activates the NADPH oxidase RESPIRATORY BURST OXIDASE homolog D (RBOHD) (Kadota et al., 2014; Li et al., 2014). NADPH oxidase uses cytosolic NADPH as reductant, electrons being transported via flavin adenine dinucleotide (FAD) and heme cofactors to the outside where oxygen is reduced to superoxide. The majority of this superoxide is assumed to dismutate very rapidly, producing hydrogen peroxide (Smirnoff and Arnaud, 2019). While RBOHD is the main NADPH oxidase isoform involved in apoplastic ROS burst, respiratory burst OXIDASE homolog F (RBOHF) peroxidase also contributes partly to ROS production during PTI (Torres et al., 2002; Zhang et al., 2007). Both isoforms participate in plant defenses against *Pseudomonas syringae* pv tomato (*Pst*) bacteria (Zhang et al., 2007; Chaouch et al., 2012; Kadota et al., 2014). Type III cell wall peroxidases, while using hydrogen peroxide to oxidatively cross-link cell wall components, can after pathogen perception also generate ROS/H_2_O_2_ (Bindschedler et al., 2006; Daudi et al., 2012). In particular, peroxidase 4 (PRX4), peroxidase 33 (PRX33), peroxidase 34 (PRX34), and peroxidase 71 (PRX71) are involved in PAMP-mediated ROS production, and PRX33/34 plays an important role in defenses against *Pst* bacteria (Daudi et al., 2012; Arnaud et al., 2017).

Although an apoplastic ROS burst is a hallmark of the early plant response to PAMPs, ROS production by chloroplasts, mitochondria, or peroxisomes has been associated with late defense responses such as effector-triggered immunity and the hypersensitive response (Camejo et al., 2016; Zechmann, 2020; Littlejohn et al., 2021). Nevertheless, the role of intracellular organelles in ROS production during PTI is emerging (Smirnoff and Arnaud, 2019; Littlejohn et al., 2021). PAMPs perception induced an inhibition of photosynthetic activity and an increase in ROS production in chloroplasts which is inhibited by bacterial effectors (Göhre et al., 2012; de Torres Zabala et al., 2015). Interestingly, crosstalk between chloroplasts, mitochondria, peroxisomes and the apoplast during PAMP-mediated ROS production have also been documented (Chaouch et al., 2012; Göhre et al., 2012; Fabro et al., 2016), and excess ROS in organelles due for example to defects in thylakoid ascorbate peroxidase in chloroplasts or catalase activity in peroxisomes activate defense gene expression through retrograde signaling and modulate defense related hormones (Chaouch et al., 2010; Camejo et al., 2016; Yuan et al., 2017; Zechmann, 2020). However, to date, a precise temporal and subcellular distribution of ROS, and particularly H_2_O_2_, during PTI has not been characterized.

The chemical probes generally used in measuring PAMP and pathogen-associated ROS production include luminol, diaminobenzidine (DAB), and 2',7’ dichlorofluorescein diacetate. These mostly lack spatial resolution and, more importantly, specificity as they can react with a range ROS such as superoxide ions (O_2_•^−^), peroxynitrite (ONOO^−^), and hydroxyl radical (OH•) (Smirnoff and Arnaud, 2019). To improve detection of H_2_O_2_, genetically encoded biosensors such as HyPer or roGFP2-Orp1, based on hydrogen peroxide-sensitive cysteine residues incorporated into fluorescent proteins, have been characterized in vitro and in vivo using diverse model organisms and are being increasingly used in plants (Schwarzländer et al., 2008; Gutscher et al., 2009; Exposito-Rodriguez et al., 2017; Müller et al., 2017; Ortega-Villasante et al., 2018; Nietzel et al., 2019; Ugalde et al., 2021). The main advantages of these sensors are the possibility to make ratiometric measurements that are independent of the level of probe expression, and the reversibility of the probe oxidation by the glutaredoxin (GRX) and glutathione (GSH) mediated reduction permitting dynamic and real-time measurements. Furthermore, given this interaction with the thiol system, probes reactive with H_2_O_2_ can be compared with those that report the redox state of the glutathione pool (Aller et al., 2013).

Recently, Arabidopsis (*Arabidopsis thaliana*) expressing roGFP2-Orp1 in the cytosol/nucleus or mitochondria has been used to study H_2_O_2_ accumulation in leaves exposed to PAMPs in mutants affected in GSH/GRX redox metabolism (Nietzel et al., 2019), showing that this probe could be successfully used to monitor the plant immune response. Here, we investigated the role of NADPH oxidases, PRXs, and upstream PTI regulators BAK1 and BIK1 on intracellular changes in H_2_O_2_/redox dynamics during PAMP-triggered immunity by crossing mutants with Arabidopsis plants expressing the cytosolic/nuclear roGFP2-Orp1 biosensor. Moreover, we generated plants expressing roGFP2-Orp1 in various subcellular compartments. Challenging leaves with *Pst* bacteria or PAMPs induced a biphasic biosensor oxidation more intense in the cytosol and nucleus than in chloroplasts, mitochondria, or peroxisomes, suggesting that the cytosol and nucleus may integrate signaling during plant defense response. Unexpectedly, our results reveal that PAMP-mediated oxidation of the cytosol/nucleus of leaves is largely independent of apoplastic ROS produced by NADPH oxidases and PRXs.

## Results

### Characterization of Arabidopsis expressing roGFP2-Orp1 and GRX1-roGFP2 in different subcellular compartments

**Figure 1:**
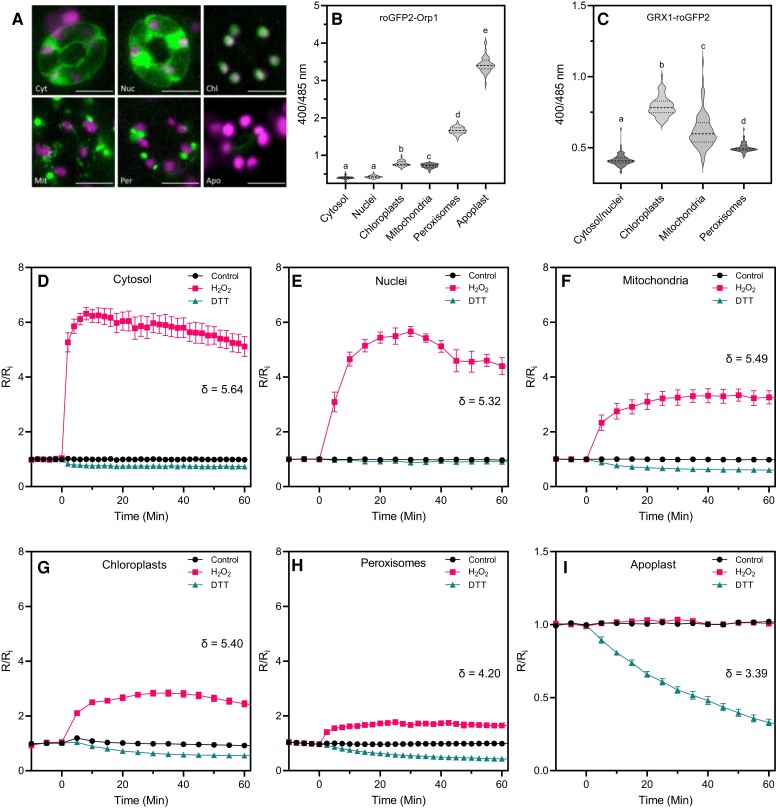
In vivo characterization of roGFP2-Orp1 targeted to different organelles. A, Subcellular localization of roGFP2-Orp1 targeted to the cytosol (Cyt), nuclei (Nuc), chloroplasts (Chl), mitochondria (Mit), peroxisomes (Per), and apoplast (Apo) in guard cells. Representative images of the fluorescence emission through a long-pass filter with a cutoff wavelength at 515 nm after excitation at 470 ± 20 nm. roGFP2-Orp1 and chloroplast fluorescence are depicted in green and magenta, respectively. Scale bars represent 10 µm. B, Initial roGFP2-Orp1 oxidation state in cytosol, nuclei, chloroplasts, mitochondria, peroxisomes, and apoplast. Horizontal lines on violin plots show the median and quartile values. Data are means of at least two independent experiments (*n* ≥ 40). Different letters indicate significant differences at *P* < 0.001 based on Tukey's HSD test. C, Initial GRX1-roGFP2 oxidation state in cytosol and nuclei, chloroplasts, mitochondria, and peroxisomes. The oxidation state of roGFP2-Orp1 and GRX1-roGFP2 (ratio 400/485 nm) in untreated condition was measured by multiwell fluorimetry (excitation at 400 ± 8 and 485 ± 8 nm; emission, 525 ± 20 nm) on leaf discs from rosette leaves of 5-week-old plants. Data are means of at least two independent experiments (*n* ≥ 40). Different letters indicate significant differences at *P* < 0.001 based on Tukey's HSD test. Horizontal lines on violin plots show the median and quartile values. D–I, In vivo characterization of organelle-targeted roGFP2-Orp1 oxidation and reduction kinetics in response to H_2_O_2_ and DTT. Leaf discs of plant expressing roGFP2-Orp1 targeted to the cytosol (D), nuclei (E), mitochondria (F), chloroplasts (G), peroxisomes (H), and apoplast (I) were exposed at *t* = 0 min to control solution, 100 mM H_2_O_2_ or 50 mM DTT. The 400/485 nm fluorescence ratio (*R*) was measured over time by multiwell fluorimetry (excitation at 400 ± 8 and 485 ± 8 nm; emission, 525 ± 20 nm) and expressed relative to the mean initial ratio (*R*_i_) before treatment (*R*/*R*_i_). Dynamic range (*δ*) of probes in each subcellular compartment was calculated from the 400/485 nm excitation ratios for the oxidized and reduced probe. Data are means ± Se from a representative experiment (*n* ≥ 4). Two-way ANOVA using repeated measures for time and Tukey's multiple comparisons analyses show H_2_O_2_ and DTT significantly (*P* < 0.01) affect *R*/*R*_i_ in all compartments except H_2_O_2_ in the apoplast (Supplemental Table S2).

To follow the dynamics of H_2_O_2_ production in different subcellular compartments during the immune response, we fused roGFP1-Orp1 to target peptides for cytosol, nuclei, chloroplasts, mitochondria, peroxisomes, and apoplast under the control of a CaMV 35S promoter (Supplemental Figure S1A and Table S1). These were expressed in Arabidopsis and their correct subcellular localization was confirmed by microscopy of leaf epidermal peels (Figure 1A, Supplemental Figure S1C). To measure thiol redox state, we used lines expressing the E_GSH_ biosensor GRX1-roGFP2 in cytosol/nucleus, chloroplasts, mitochondria, or peroxisomes (Marty et al., 2009; Rosenwasser et al., 2011; Park et al., 2013; Albrecht et al., 2014). All the lines used here had no visible defects in growth or development (Supplemental Figure S1B), although, in another study, expression of roGFP2-Orp1 in the mitochondrial matrix using a different target peptide caused dwarfism (Nietzel et al., 2019). The ratio of fluorescence emission (505–545 nm) with excitation at 400 and 485 nm was measured in leaf discs. The 400/485 ratio increases when the probe is oxidized. roGFP2-Orp1 was more oxidized in the organelles than the cytosol or nucleus and was highly oxidized in the apoplast (Figure 1B). The oxidation state of GRX1-roGFP2 targeted to the cytosol/nucleus, chloroplasts, and mitochondria was similar to the corresponding roGFP2-Orp1 reporters although the peroxisomal GRX1-roGFP2 was relatively more reduced (Figure 1C). The dynamic range (DR) (Nietzel et al., 2019) of roGFP2-Orp1 oxidation and reduction in organelles was investigated by treating leaf discs with 100 mM H_2_O_2_ and 50 mM dithiothreitol (DTT) (Figure 1D–I). The dynamic range values are shown for each subcellular location (Figure 1D–I). According to the initial oxidation state, the response to H_2_O_2_ was stronger in the cytosol and nuclei than in other organelles.

Conversely, roGFP2-Orp1 was more reduced by DTT in chloroplasts, mitochondria, and peroxisomes than in cytosol and nuclei. According to the 400/485 nm ratio, the degree of probe oxidation was stronger in the apoplast (96.5%), peroxisomes (69.9%), mitochondria (35.3%), chloroplasts (32%) than in cytosol (6.7%) and nuclei (6.2%). This results in a lower dynamic range for peroxisomal and especially apoplastic roGFP2-Orp1 compared to cytosolic, nuclear, chloroplastic, and mitochondrial roGFP2-Orp1 (Figure 1D–I). Therefore, the roGFP2-Orp1 targeted to the apoplast was excluded from further analyses because it is fully oxidized in control conditions (Figure 1I). The dose–response of roGFP2-Orp1 to exogenous H_2_O_2_ between 10 µM and 100 mM was determined in a cytosol/nucleus expressing line (Nietzel et al., 2019) and 100 µM H_2_O_2_ was sufficient to detectably oxidize the probe (Supplemental Figure S2, A and B).

**Figure 2:**
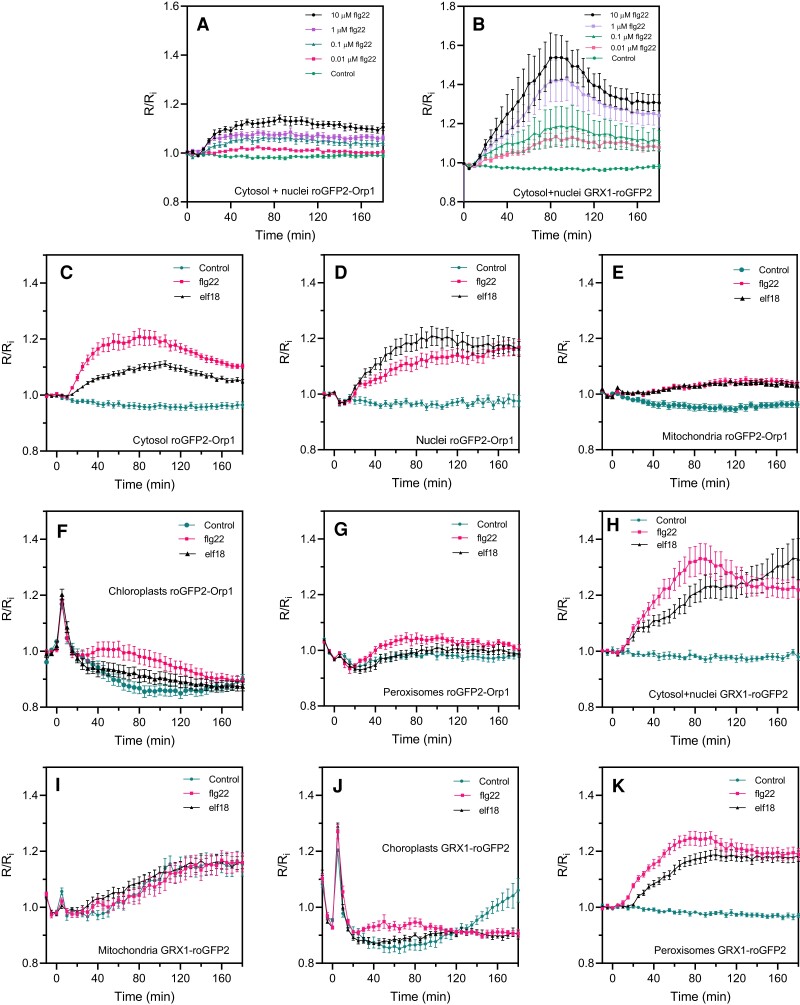
PAMP-induced roGFP2-Orp1 and GRX1-roGFP2 redox dynamics in different subcellular compartments. Dose–response kinetics of cytosolic/nuclear roGFP2-Orp1 (A) and GRX1-roGFP2 (B) oxidation in leaves in response to the PAMP flg22. Leaf discs were exposed at *t* = 0 min to control solution or different concentrations of flg22. The 400/485 nm fluorescence ratio (*R*) was measured over time by multiwell fluorimetry and expressed relative to the mean initial ratio (*R*_i_) before treatment. Data are means ± Se from a representative experiment (*n* = 6). The experiments have been repeated at least twice with similar results. Kinetics of roGFP2-Orp1 oxidation in the cytosol (C), nuclei (D), mitochondria (E), chloroplasts (F), and peroxisomes (G) and GRX1-roGFP2 oxidation in the cytosol and nuclei (H), mitochondria (I), chloroplasts (J), and peroxisomes (K) in response to PAMPs. Leaf discs (C–K) were exposed at *t* = 0 min to control solution, 1 µM elf18, or 1 µM flg22. The 400/485 nm fluorescence ratio (*R*) was measured over time by multiwell fluorimetry and expressed relative to the mean initial ratio (*R*_i_) before treatment. Data are means ± Se from two independent experiments (*n* ≥ 6, C–F, H, I, K) or a representative experiment (*n* ≥ 5, G and J). Two-way ANOVA using repeated measures for time and Tukey's multiple comparisons analyses are shown in Supplemental Table S2. All treatments were significantly different from control (*P* < 0.05) except: B (0.1 µM flg22); F, G (flg22); I, The Rred/Ri and Rox/Ri fluorescence ratios for fully reduced/fully oxidized probes in each compartment (Figure 1) were: cytosol 0.89/5.05; nuclei 0.89/4.76; mitochondria 0.61/3.35; chloroplasts 0.53/2.88, and peroxisomes 0.43/1.79.

To investigate the oxidative events during PAMP-triggered immunity, we analyzed the responsiveness of cytosol/nucleus-localized roGFP2-Orp1 to PAMP treatments. roGFP2-Orp1 was oxidized by the PAMP flagellin 22 (flg22) at a concentration as low as 0.01 µM (Figure 2A), which is comparable to other physiological assays using this elicitor (Felix et al., 1999). Probe oxidation started after a lag of 10 min and reached a plateau at 60 min, remaining oxidized over the 180 min duration of the experiment. As a control, mutated flg22 peptide did not affect roGFP2-Orp1 oxidation (Supplemental Figure S2C). GRX1-roGFP was similarly oxidized but with a somewhat larger change compared to roGFP-Orp1 (Figure 2B). The PAMP elf18 peptide at 1 μM elicited a similar roGFP2-Orp1 oxidation response to flg22 (Supplemental Figure S3).

roGFP2-Orp1 is also oxidized in vitro by peroxynitrite (ONOO^−^) but not by nitric oxide (NO) (Müller et al., 2017). Peroxynitrite is formed from NO and superoxide (O_2_^−^), which are both produced during plant defense responses and programmed cell death (Vandelle and Delledonne, 2011; Wilkins et al., 2011). We therefore tested the response of the roGFP2-Orp1 and GRX1-roGFP2 probes to the NO donor sodium nitroprusside (SNP) and the NO scavenger carboxyphenyl-4,4,5,5-tetramethylimidazoline-1-oxyl 3-oxide (cPTIO) (Delledonne et al., 1998). High SNP concentration and, surprisingly, cPTIO caused probe oxidation (Supplemental Figure S4). However, the flg22-induced roGFP2-Orp1 oxidation was not affected by cPTIO (Supplemental Figure S4B) while GRX1-roGFP2 oxidation was moderately decreased by cPTIO at later time points (Supplemental Figure S4D). These results suggest that the increase in roGFP2-Orp1 oxidation by flg22 reflects mainly the production of H_2_O_2_ rather than NO or ONOO^−^. Alternatively, flg22 could impair biosensor re-reduction. Overall, these results indicate the suitability of roGFP2-Orp1 and GRX1-roGFP2 to measure stimulus-driven responses in all subcellular compartments.

### PAMP-mediated roGFP2-Orp1 and GRX1-roGFP2 oxidation is stronger and faster in the cytosol and nucleus than in other organelles

To understand how the activation of the immune response modulates oxidation state in different subcellular compartments, we treated leaf discs expressing roGFP2-Orp1 targeted to the cytosol, nucleus, chloroplasts, mitochondria, or peroxisomes with the PAMPs flg22 and elf18 and measured the 400/485 nm fluorescence ratio (expressed relative to the initial value: *R*/*R*_i_) over 180 min (Figure 2C–G). PAMP-induced oxidation of roGFP2-Orp1 targeted to the nucleus or cytosol was strong, starting early from 10 min after treatment (Figure 2C). Brief exposure of all the samples to light (photon flux density [PPFD]∼ 10 µmol m^−2^ s^−1^) during the PAMP treatments caused a transient oxidation of organelle-localized probes and this was followed by a smaller PAMP-induced oxidation than in the cytosol and nucleus (Figure 2C–G). The light-induced roGFP2-Orp1 oxidation was particularly strong in chloroplasts (Figure 2F). The responses to elf18 and flg22 were similar in mitochondria while in the other compartments flg22 was more effective than elf18 in oxidizing roGFP2-Orp1 (Figure 2C–G). In conclusion, the cytosol and nucleus showed faster, stronger, and more prolonged PAMP-mediated roGFP2-Orp1 oxidation than the other compartments. GRX1-roGFP2 targeted to the cytosol/nuclei or chloroplasts showed similar profiles to their roGFP2-Orp1 counterparts (Figure 2H–K). In contrast, there was no difference between control and PAMP treatments for mitochondrial GRX1-roGFP2 (Figure 2I). In peroxisomes, GRX1-roGFP2 was more strongly oxidized by PAMPs compared to roGFP2-Orp1 (Figure 2K).

### Hydrogen peroxide-induced roGFP2-Orp1 oxidation is influenced by the NADPH oxidase RBOHF, apoplastic peroxidases PRX4, PRX33, and PRX34 and vitamin C defective 2 (VTC2)

**Figure 3:**
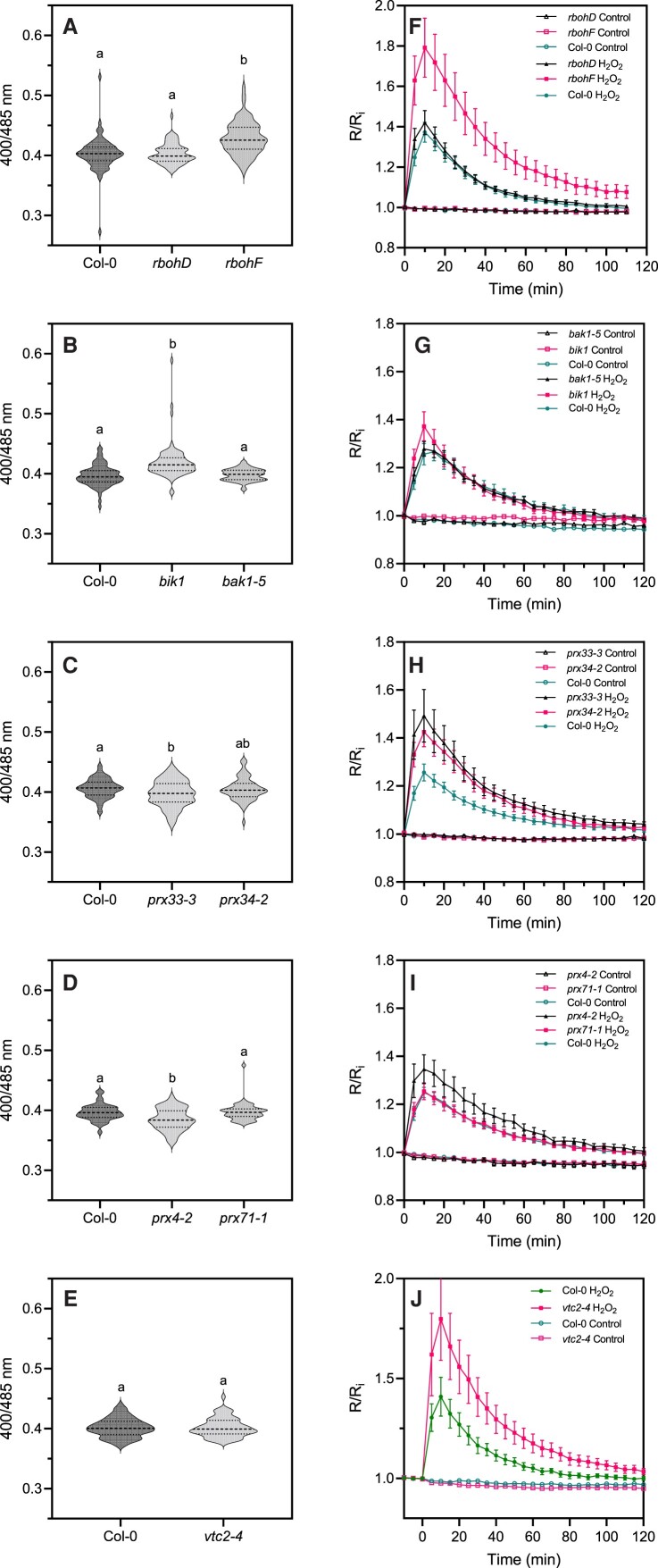
Initial oxidation state and H_2_O_2_-induced oxidation of roGFP2-Orp1 in mutants of PTI regulators, NADPH oxidases, and apoplastic peroxidases. Initial cytosolic/nuclear roGFP2-Orp1 oxidation state in *rbohD* and *rbohF* (A), *bak1-5* and *bik1* (B), *prx33-3* and *prx34-2* (C), *prx4-2* and *prx71-1* (D) and *vtc2-4* (E) mutants. The oxidation state of cytosolic/nuclear roGFP2-Orp1 (ratio 400/485 nm) in untreated conditions was measured by multiwell fluorimetry on leaf discs. Data are means ± Se (Figures H–J) or violin plots with horizontal lines showing the median and quartile values (Figures C–E) from at least three independent experiments (*n* ≥ 40). Different letters indicate significant differences at *P* < 0.001 (A, B, and E), *P* < 0.05 (C), and *P* < 0.01 (D) based on Tukey's HSD test. Kinetics of cytosolic/nuclear roGFP2-Orp1 oxidation in leaves of *rbohD* and *rbohF* (F), *bak1-5* and *bik1* (G), *prx33-3* and *prx34-2* (H), *prx4-2* and *prx71-1* (I), and *vtc2-4* (J) mutants in response to exogenous H_2_O_2_. Leaf discs were exposed at *t* = 0 min to control solution or 1 mM H_2_O_2_, the 400/485 nm fluorescence ratio (*R*) was measured over time by multiwell fluorimetry and expressed relative to the mean initial ratio (*R*_i_) before treatment. Data are means ± Se from three independent experiments (*n* ≥ 15, F–I) or a representative experiment (*n* ≥ 6, J). Two-way ANOVA using repeated measures for time and Tukey's multiple comparisons analyses are shown in Supplemental Table S2. H_2_O_2_ significantly (*P* < 0.05) increased probe oxidation in all cases. *rbohF* was significantly (*P* < 0.05) more oxidized than Col-0. The Rred/Ri and Rox/Ri fluorescence ratios for fully reduced/fully oxidized probes in the cytosol/nuclei (Supplemental Figure S2) were 0.85/5.70.

Arabidopsis expressing cytosol/nucleus-localized roGFP2-Orp1 (Nietzel et al., 2019) was crossed with mutants affected in upstream regulators of plant immunity BAK1 and BIK1 (Chinchilla et al., 2007; Lu et al., 2010; Zhang et al., 2010) and enzymes involved in apoplastic ROS production such as the NADPH oxidases RBOHD and RBOHF (Torres et al., 2002; Zhang et al., 2007; Chaouch et al., 2012) and the apoplastic peroxidases PRX4, PRX33, PRX34, and PRX71 (Daudi et al., 2012; Arnaud et al., 2017). In addition, roGFP2-Orp1 was introduced into *vtc2-4*, which has an 80% reduction of ascorbate concentration (Lim et al., 2016). Note that contrary to the other lines used which are knock-out mutants, the *prx4-2* mutation in the 3'UTR induces overexpression of PRX4 (Arnaud et al., 2017) and the *bak1-5* mutant is specifically impaired in PTI responses due to a mis-sense nucleotide substitution (Schwessinger et al., 2011). In untreated leaf discs, roGFP2-Orp1 was unexpectedly more oxidized in *rbohF* and *bik1* mutant backgrounds than in Col-0 wild-type (WT), while the oxidation state of the biosensor was unaffected in *bak1-5*, *rbohD*, and *vtc2-4* mutants (Figure 3A–E). This suggests a lower antioxidant capacity in the *bik1* and *rbohF* mutants, possibly due to long-term adaptation to the lack of superoxide production by RBOHF. Low ascorbate in *vtc2-4* did not affect roGFP2-Orp1 oxidation in our growing conditions until addition of H_2_O_2_ (Figure 3E). By contrast, roGFP2-Orp1 was significantly more reduced in *prx33-3* than WT suggesting that PRX33 produces H_2_O_2_ even in unstressed conditions (Figure 3C). On the contrary, the stronger reduction of roGFP2-Orp1 in the *prx4-2* background (Figure 3D) indicates that PRX4 scavenges H_2_O_2_ (Francoz et al., 2015). Differences in the extent of roGFP2-Orp1 oxidation, depending on the mutant backgrounds prompted an investigation of the effect of exogenous H_2_O_2_. Compared to WT, roGFP2-Orp1 became more oxidized in *vtc2-4*, *prx4-2* (an overexpression line), *prx33-3, prx34-2*, and particularly *rbohF*, in response to 1 mM H_2_O_2_ (Figure 3F–J). The hypersensitivity of *prx33-3* and *prx34-2* mutants to H_2_O_2_ could be explained by a defect in H_2_O_2_ removal, as these apoplastic peroxidases are also known to consume H_2_O_2_ to catalyze the oxidation of monolignols for lignin polymerization (Demont-Caulet et al., 2010). Moreover, this result confirms that ascorbate is important in removing excess H_2_O_2_.

### A PAMP-induced RBOHD-independent but BAK1/BIK1-dependent roGFP2-Orp1 oxidation follows the early oxidative burst detected by luminol

**Figure 4:**
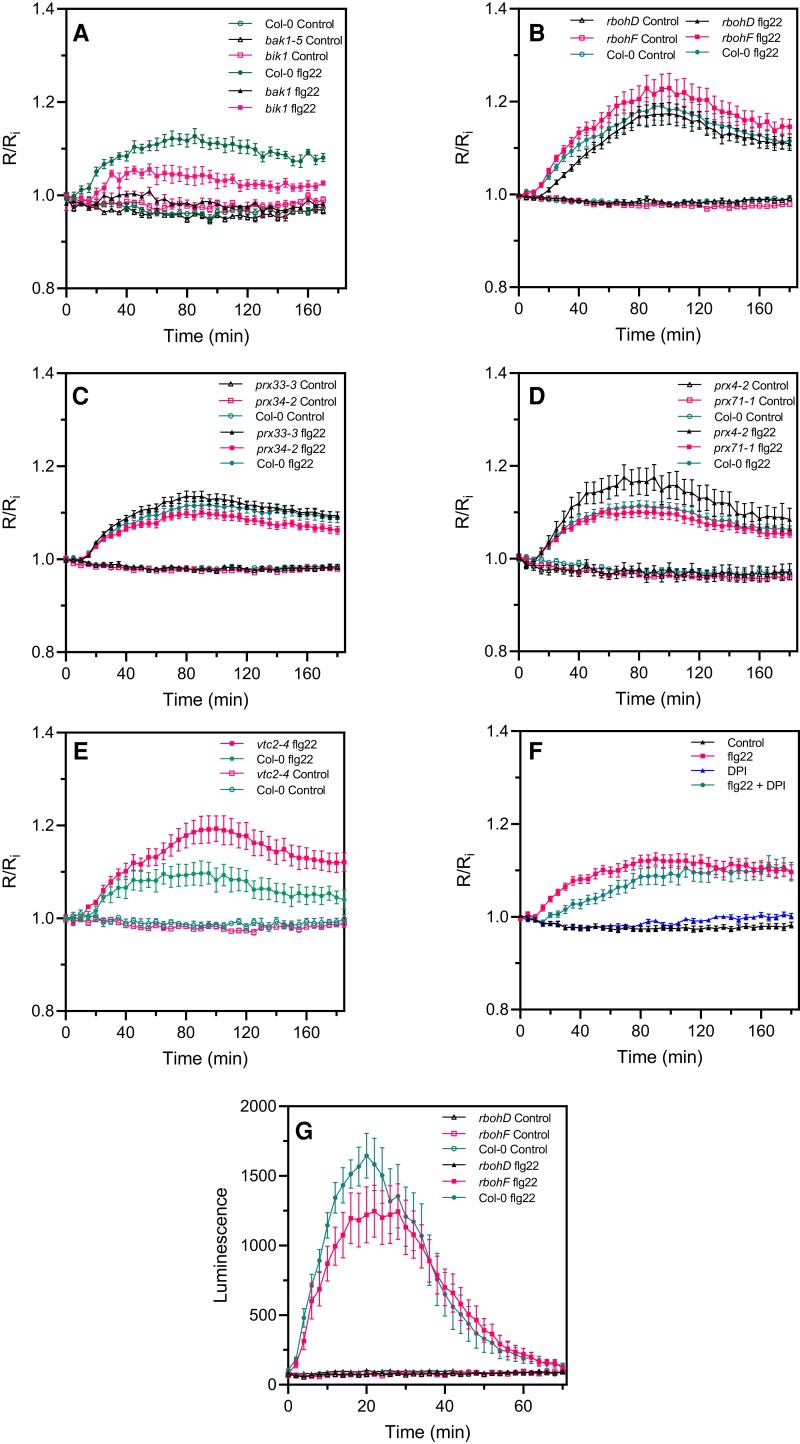
flg22-induced intracellular roGFP2-Orp1 redox dynamics in mutants affecting PTI-mediated ROS production in the apoplast. Kinetics of cytosolic/nuclear roGFP2-Orp1 oxidation in leaves of *bak1-5* and *bik1* (A), *rbohD* and *rbohF* (B), *prx33-3* and *prx34-2* (C), *prx4-2* and *prx71-1* (D) and *vtc2-4* (E), mutants in response to the PAMP flg22. Leaf discs were exposed at *t* = 0 min to control solution or 1 µM flg22. The ratio 400/485 nm (*R*) was measured over time by multiwell fluorimetry and expressed relative to the mean initial ratio (*R*_i_) before treatment (*R*/*R*_i_). Data are means ± Se from three independent experiments (*n* ≥ 15, B–D). In (A and E), a representative experiment is shown (*n* = 6). F, Effect of DPI on flg22-induced oxidation of roGFP2-Orp1. After 2 h of pretreatment with control solution or 20 µM DPI, leaf discs from Col-0 were exposed at *t* = 0 min to control solution or 1 µM flg22. The 400/485 nm fluorescence ratio (*R*) was measured over time by multiwell fluorimetry and expressed relative to the mean initial ratio (*R*_i_) before flg22 treatment. Data are means ± Se of three independent experiments (*n* ≥ 10). G, PAMP-induced apoplastic ROS production detected by luminol assay in Col-0 WT, *rbohD*, and *rbohF* mutants. The luminescence was measured over time after treatment with control solution or 1 µM flg22 at *t* = 0 min. Data are means ± Se (*n* = 6) from a representative experiment. In (A–G), two-way ANOVA using repeated measures for time and Tukey's multiple comparisons analyses are shown in Supplemental Table S2. flg22 significantly (*P* < 0.05) increased probe oxidation in all mutants except *bak1* (A). flg22-treated *bik1* and *vtc2-4* flg22 were significantly different from flg22-treated Col-0 (A and E). flg22-induced oxidation was significantly decreased by DPI (F). The 400/485 nm fluorescence ratios for fully reduced/fully oxidized probes in the cytosol/nuclei (Figure 1) were 0.85/5.70.

The *bak1-5*, *bik1, rbohD, rbohF, prx4-2*, *prx33-3*, *prx34-2*, *prx71-1*, and *vtc2-4* mutants were subjected to flg22 or elf18 treatments. As expected, the *vtc2-4* mutant was hypersensitive to flg22 (Figure 4E) indicating that ascorbate is necessary for controlling excess H_2_O_2_ during the immune response. The *bik1* and *bak1-5* mutants were strongly impaired in PAMP-triggered roGFP2-Orp1 oxidation (Figure 4A). *rbohD* had a significantly delayed PAMP response, not reaching the oxidation level of Col-0 until 50 or 70 min after addition of either flg22 (Figure 4B) or elf18 (Supplemental Figure S4A), respectively. Correspondingly, pretreatment with the nonspecific flavoenzyme/NADPH oxidase inhibitor diphenyleneiodonium chloride (DPI) mimicked the response of *rbohD* by causing a significant lag in flg22-induced roGFP2-Orp1 oxidation (Figure 4F). roGFP-Orp1 oxidation kinetics were compared to the PAMP-induced apoplastic oxidative burst using a luminol assay (Felix et al., 1999). This oxidative burst showed the expected fast and transient response, peaking at 20 min post flg22 addition, and was absent in *rbohD* (Figure 4G). Therefore, the delayed roGFP-Orp1 oxidation in *rbohD* corresponds to the time of the “missing” apoplastic burst. By contrast, *rbohF*, *prx33-3*, and *prx71-1* were not affected in PAMP-induced roGFP2-Orp1 oxidation while the *prx34-2* mutant exhibited a slight reduction of roGFP2-Orp1 oxidation from 100 min after PAMP treatment (Figure 4B–D and Supplemental Figure S4A–C). The *prx4-2* overexpression line showed an enhanced oxidation of roGFP2-Orp1 after PAMP (and H_2_O_2_) treatments (Figure 4D), suggesting that PRX4 can produce H_2_O_2_ under stress conditions that over-accumulates in the cytosol.

### Complex transcriptional changes of antioxidant genes following PAMP or bacteria challenge

Changes in oxidation of cytosolic roGFP2-Orp1 result from the antagonistic impacts of H_2_O_2_ production and removal. We therefore used publicly available microarray data to analyze changes in expression profile of genes coding for antioxidant enzymes in response to PAMP treatment and infection with *Pseudomonas syringae* pv tomato (*Pst*) DC3000 bacteria, together with their predicted or confirmed subcellular localization (Supplemental Figure S5). In general, the expression of genes encoding chloroplastic superoxide dismutase (SOD), monodehydroascorbate reductase (MDAR), dehydroascorbate reductase (DHAR), glutathione peroxidase (GPX), and peroxiredoxin (PrxR) genes is down-regulated while cytosolic MDAR and GPX expression are upregulated after PAMP and bacteria treatment. Interestingly, about half of genes encoding *thioredoxins* (Trx) whatever their predicted subcellular localization (cytosol or chloroplast) are down-regulated by PAMPs and bacteria (Supplemental Figure S5), and a significant number of cytosolic/plasma membrane localized glutaredoxin (GLR) genes are down-regulated by PAMP or the disarmed *Pst hrcC* strain defective in the type 3 secretion (T3S) effector system. Notably, some of these down-regulated *GRX* genes are instead upregulated by WT *Pst* DC3000 bacteria suggesting that bacterial effectors may upregulate their transcription.

Few antioxidant genes (only approximately 20%) showed significant changes of expression an hour after flg22 treatment. We therefore directly analyzed the enzymatic activities of ascorbate peroxidases (APX) and catalases (CAT) that play a major role in intracellular H_2_O_2_ removal (Smirnoff and Arnaud, 2019). APX and CAT activities were not affected after 2 h of flg22 treatment in Col-0, roGFP2-Orp1, roGFP2-Orp1 *bak1-5*, and roGFP2-Orp1 *rbohD* (Supplemental Figure S6).

### A second large oxidation of cytosolic roGFP2-Orp1 and GRX1-roGFP2 in response to *Pseudomonas syringae* and PAMPs

**Figure 5:**
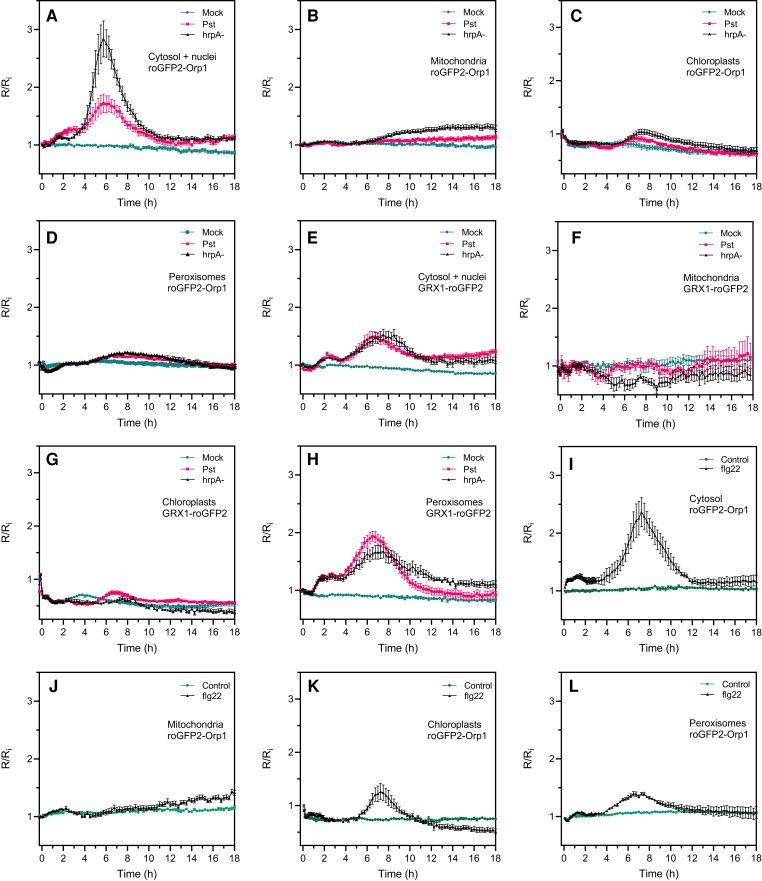
*Pseudomonas syringae* bacteria and flg22 induce a biphasic roGFP2-Orp1 and GRX1-roGFP2 oxidation in the cytosol. Oxidation kinetics of roGFP2-Orp1 targeted to the cytosol/nuclei (A), mitochondria (B), chloroplasts (C), and peroxisomes (D), and GRX1-roGFP2 targeted to the cytosol/nuclei (E), mitochondria (F), chloroplasts (G), and peroxisomes (H) in response to WT *Pst* DC3000 and disarmed *Pst hrpA* bacteria. Leaf discs were exposed at *t* = 0 min to mock (10 mM MgCl_2_), 10^8^ cfu mL^−1^*Pst* DC3000 (*Pst*), or 10^8^ cfu mL^−1^*Pst hrpA* bacteria. Long-term oxidation kinetics of roGFP2-Orp1 targeted to the cytosol (I), mitochondria (J), chloroplasts (K), and peroxisomes (L) in response to PAMP. Leaf discs were exposed at *t* = 0 min to control solution or 1 µM flg22. In (A–L), the 400/485 nm fluorescence ratio (*R*) was measured over time by multiwell fluorimetry and expressed relative to the mean initial ratio (*R*_i_) before treatment. Data are means ± Se from two independent experiments (*n* ≥ 8, C, D, F, and G) or a representative experiment (*n* ≥ 5, A, B, E, and H–L). Two-way ANOVA using repeated measures for time and Tukey's multiple comparisons analyses are shown in Supplemental Table S2. *Pst* DC3000 and *Pst hrpA* significantly (*P* < 0.05) increased roGFP2-Orp1 oxidation in cytosol (A) and nuclei (B) and GRX1-roGFP2 oxidation in cytosol/nuclei (E) and peroxisomes (H). *Pst* DC3000 and *Pst hrpA* were significantly (*P* < 0.05) different in roGFP2-Orp1 oxidation in cytosol (A). flg22 significantly (*P* < 0.05) increased roGFP2-Orp1 oxidation in cytosol (H), chloroplasts (K), and peroxisomes (L). The 400/485 nm fluorescence ratios for fully reduced/fully oxidized probes in each compartment (Figure 1) were: cytosol 0.89/5.08; nuclei 0.98/4.76; mitochondria 0.62/3.35; chloroplasts 0.53/2.88, and peroxisomes 0.43/1.79.

We investigated the response of organelle-targeted roGFP2-Orp1 and GRX1-roGFP2 to *Pst* DC3000 WT bacteria and the type 3 secretion (T3S)-deficient *Pst hrpA* mutant strain that fails to secrete effectors (Figure 5). Because no oxidation of the sensors was observed up to 18 h after inoculation when bacteria were simply added to the wells, leaf discs were vacuum infiltrated to facilitate the penetration of bacteria. As with the PAMP treatment, we observed an early increase of biosensor oxidation in the cytosol and nucleus from 1 h after inoculation that remained stable up to 3 h (Figure 5A). Interestingly, a second very strong increase of roGFP2-Orp1 (cytosol/nucleus or cytosol) oxidation occurred from 4 h and peaking at 6 h post-inoculation. Subsequently, roGFP2-Orp1 oxidation state returned to first early phase level at 11 h post-inoculation (Figure 5A). The amplitude of this second oxidation was much higher for *Pst hrpA* than with WT *Pst* bacteria suggesting that *Pst* bacteria secrete effectors to counteract oxidation of the cytosol (Figure 5A). On the contrary, no significant increase in roGFP2-Orp1 oxidation was observed in chloroplasts, mitochondria, and peroxisomes during the first phase up to 6–8 h after *Pst* DC3000 or *Pst hrpA* challenge (Figure 5B–D). Only a slight increase in oxidation was observed from 6 h in chloroplasts and peroxisomes followed by a decrease in oxidation level from 8 to 10 h reaching control levels at the end of the experiment (Figure 5B–D). In mitochondria, the roGFP2-Orp1 oxidation was weak compared to the cytosol/nucleus, occurred later from 8 h post-inoculation, and remained stable over time (Figure 5B). roGFP2-Orp1 oxidation in chloroplasts and mitochondria was higher after treatment with *Pst hrpA* than WT *Pst* DC3000 while in peroxisomes both bacteria induced a similar response (Figure 5D). The response to bacteria of GRX1-roGFP2 in the cytosol/nucleus and in peroxisomes was somewhat like cytosolic/nuclear roGFP2-Orp1 with two phases of increase in E_GSH_, the second stronger than the first one (Figure 5E–H). However, the increase in GRX1-roGFP2 oxidation was similar for *Pst* WT and *Pst hrpA* bacteria indicating that bacterial effectors did not influence E_GSH_. Surprisingly, in chloroplasts, bacteria induced a decrease in GRX1-roGFP2 oxidation compared to mock treatment during the first phase from 2 to 5 h after bacteria inoculation (Figure 5G). Following this, *Pst* DC3000 bacteria, but not the *Pst hrpA* strain, induced a slight increase in chloroplast GRX1-roGFP2 oxidation at 5 h that decreased slowly from 7 h post-inoculation. In mitochondria, the bacteria did not change the roGFP2-GRX1 oxidation state (Figure 5F) as observed earlier for PAMP treatment.

As a comparison, we analyzed the long-term effects of flg22 on the oxidation of cytosolic, chloroplastic, mitochondrial, and peroxisomal roGFP2-Orp1 after vacuum infiltration of leaf discs (Figure 5I–L). Similar results were obtained for the first phase of PAMP-mediated roGFP2-Orp1 oxidation compared to noninfiltrated leaf discs (Figure 2), with a stronger oxidation of roGFP2-Orp1 in the cytosol than in chloroplasts, mitochondria, and peroxisomes (Figure 5I–L). As for *Pst hrpA* bacteria inoculation, flg22 triggered a second massive phase of roGFP2-Orp1 oxidation in the cytosol (Figure 5I), but also to a lesser extent in chloroplasts and peroxisomes (Figure 5, K and L). This second increase started later in chloroplast (5 h) compared to cytosol and peroxisomes (4 h) and ended earlier in chloroplasts and peroxisomes (10 h) than in cytosol (12 h). In mitochondria, the second increase in flg22-mediated roGFP2-Orp1 oxidation was weak, occurred later at 10 h post-treatment and was constant up to 18 h (Figure 5J). Altogether, these results suggest that bacteria or PAMPs induced a first phase of roGFP2-Orp1 oxidation in cytosol/nucleus with no major impact on roGFP2-Orp1 oxidation in other organelles, but the second phase of roGFP2-Orp1 oxidation in the cytosol was larger and followed with some delay an increase in roGFP2-Orp1 oxidation in chloroplasts, mitochondria, and peroxisomes.

### The second cytosolic oxidation induced by PAMPs or bacteria is not dependent on RBOHD

**Figure 6:**
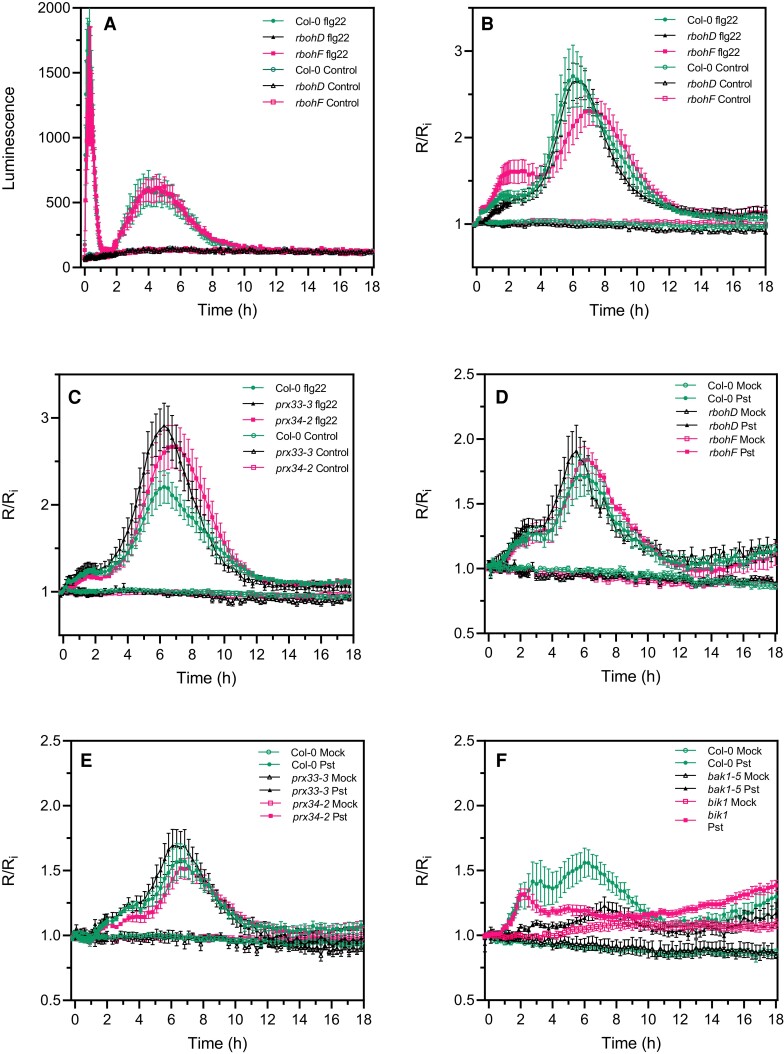
The second PAMP- and bacteria-triggered roGFP2-Orp1 oxidation event in the cytosol is not affected in *rbohD*, *rbohF*, *prx33-3*, and *prx34-2* mutants. A, Long-term kinetics of PAMP-induced apoplastic ROS production detected by luminol assay in Col-0 WT, *rbohD*, and *rbohF* mutants. The luminescence was measured over time after treatment with control solution or 1 µM flg22 at *t* = 0 min. Data are means ± Se (*n* = 5) from a representative experiment. Long-term kinetics of roGFP2-Orp1 oxidation in leaves of *rbohD* and *rbohF* (B) and *prx33-3* and *prx34-2* (C) mutants in response to flg22. Leaf discs were exposed at *t* = 0 min to control solution or 1 µM flg22. Long-term kinetics of roGFP2-Orp1 oxidation in leaves of *rbohD* and *rbohF* (D), *prx33-3* and *prx34-2* (E), and *bak1-5* and *bik1* (F) mutants in response to WT *Pst* DC3000 bacteria. Leaf discs were exposed at *t* = 0 min to Mock control (10 mM MgCl_2_) or 10^8^ cfu mL^−1^*Pst* DC3000 (*Pst*) bacteria. In (B–F), the 400/485 nm fluorescence ratio (*R*) was measured over time by multiwell fluorimetry and expressed relative to the mean initial ratio (*R*_i_) before treatment. Data are means ± Se from two independent experiments (*n* ≥ 7, B–C, E) or a representative experiment (*n* ≥ 4, D and F). Two-way ANOVA using repeated measures for time and Tukey's multiple comparisons analyses are shown in Supplemental Table S2. Pst-treated *rbohD*, *rbohF*, *prx33-3*, and *prx34-2* mutants were not significantly different to Pst-treated Col-0 (A–E). Pst-treated *bak1* was not significantly different to mock *bak1-5*. Pst-treated *bak1-5* and *bik1* were significantly different (*P* < 0.05) to Pst-treated Col-0. Pst-treated *rbohD*, *rbohF*, *prx33-3*, and *prx34-2* mutants were not significantly different to Pst-treated Col-0 (A–E). Pst-treated *bak1* was not significantly different to mock *bak1-5*. Pst-treated *bak1-5* and *bik1* were significantly different (*P* < 0.05) to Pst-treated Col-0. The 400/485 nm fluorescence ratios for fully reduced/fully oxidized probes in the cytosol/nuclei (Figure 1) were 0.85/5.70.

While a second apoplastic ROS burst was for a long time attributed to effector-triggered immunity, it was shown recently using luminol assays that PTI can induce a second apoplastic ROS burst of lower (flg22) or higher (lipopolysaccharide) amplitude compared to the first apoplastic burst (Shang-Guan et al., 2018; Ngou et al., 2021; Yuan et al., 2021). We confirmed that flg22 induced a second apoplastic ROS burst, measured by luminol oxidation, peaking at 4 h after treatment and three times less intense but more prolonged than the first ROS burst (Figure 6A). Therefore, a PAMP-triggered apoplastic ROS burst precedes roGFP2-Orp1 oxidation in the cytosol for both phases of ROS production. Interestingly, the *rbohD* mutant was completely impaired in PAMP-mediated apoplastic ROS production during the first and the second ROS bursts while the *rbohF* mutant showed WT apoplastic ROS production after flg22 treatment (Figure 6A). We analyzed the second phase of PAMP- and bacteria-triggered cytosolic roGFP2-Orp1 oxidation in the *rbohD*, *rbohF*, *prx33-3*, and *prx34-2* mutants (Figure 6, B and C). These mutants exhibited WT biphasic roGFP2-Orp1 oxidation in the cytosol after flg22 treatment (Figure 6, B and C). Compared to Col-0, the slight increase in flg22-triggered roGFP2-Orp1 oxidation observed in the *rbohF* mutant during the first phase (Figure 6B) or the slight increase in roGFP2-Orp1 oxidation in *prx33-3* and *prx34-2* mutants during the second phase (Figure 6C) were not statistically significant. Similar results were obtained after *Pst* DC3000 inoculation with no significant differences in roGFP2-Orp1 oxidation for both phases of roGFP2-Orp1 oxidation between Col-0 WT and the *rbohD*, *rbohF*, *prx33-3*, and *prx34-2* mutants (Figure 6, D and E). Interestingly, the *bak1-5* mutant was defective in bacteria-triggered H_2_O_2_ production during the first and second phases while the *bik1* mutant was impaired in roGFP2-Orp1 oxidation only during the second phase of roGFP2-Orp1 oxidation (Figure 6F).

These results suggest that the second phase of roGFP2-Orp1 oxidation in the cytosol during the immune response requires the PTI regulators BAK1 and BIK1 but is independent of apoplastic ROS production mediated by the NADPH oxidases RBOHD and RBOHF or the peroxidases PRX33 and PRX34.

## Discussion

### The use of roGFP2-Orp1 and GRX1-roGFP2 to measure oxidative events in response to PAMPs and *Pseudomonas syringae*

**Figure 7:**
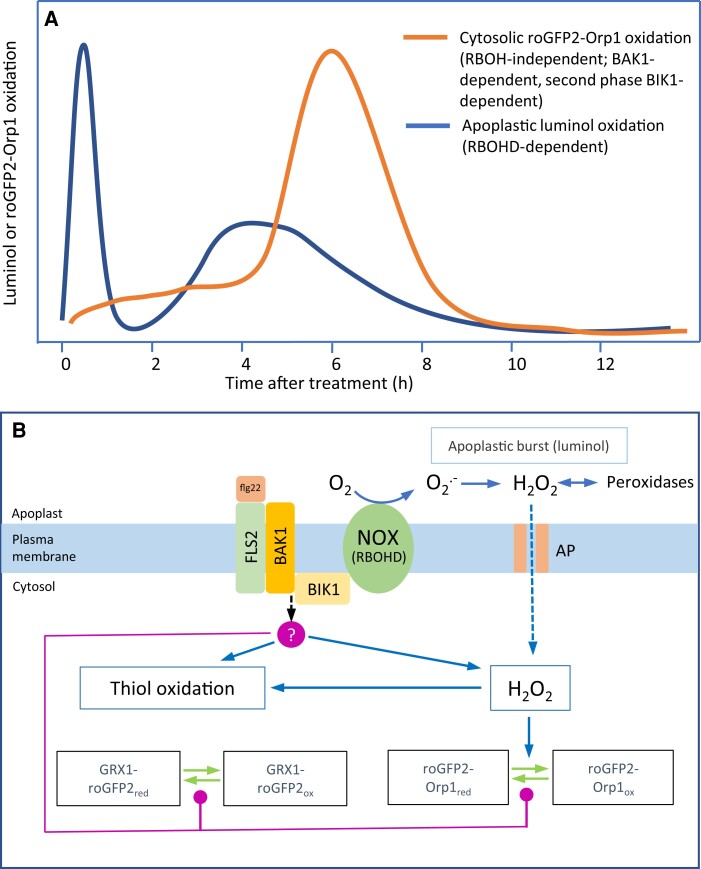
A model of apoplastic and cytosolic oxidative events during the immune response. PAMPs and virulent *Pseudomonas syringae* cause distinct oxidative events focused, respectively, on the apoplast and cytosol. The apoplastic oxidation has a very rapid large burst followed by a smaller burst at 4 h (A). Both are dependent upon the RBOHD NADPH oxidase isoform and associated with relatively small cytosolic oxidation. In contrast, oxidation of the cytosol-localized biosensors roGFP2-Orp1 and GRX1-roGFP2 starts after the initial apoplastic burst and is followed by a large oxidation event peaking at 6 h (A). This oxidation is independent of RBOHD but dependent on BAK1, the flagellin co-receptor. The second phase is also dependent on the BIK1 kinase and is suppressed by *Pseudomonas syringae* effectors. roGFP2-Orp1 is H_2_O_2_-specific (B), so oxidation could indicate increased H_2_O_2_ production in the cytosol. GRX1-roGFP2 oxidation may follow because of H_2_O_2_-induced thiol (glutathione) oxidation. BIK1 or BAK1 could activate an unidentified cytosolic H_2_O_2_ production mechanism or decrease H_2_O_2_ scavenging capacity (B, red circle). Alternatively, BIK1 or BAK1 could mediate decreased activity of thiol/glutaredoxin-based reduction of the biosensors (B, red lines). AP, H_2_O_2_ permeable aquaporin; NOX, NADPH oxidase.

We produced Arabidopsis plants expressing the H_2_O_2_ sensitive biosensor roGFP2-Orp1 targeted to cytosol, nuclei, chloroplasts, mitochondria, and peroxisomes and used these to investigate the kinetics and origin of the oxidative burst during the immune response of Arabidopsis. We compared roGFP2-Orp1 to previously produced GRX1-roGFP2 glutathione redox potential (E_GSH_) probe in cytosol/nucleus, chloroplasts, mitochondria, and peroxisomes (Gutscher et al., 2008; Rosenwasser et al., 2011; Park et al., 2013; Albrecht et al., 2014). Across the various treatments, the responses of roGFP2-Orp1 and GRX1-roGFP2 were very similar, although in a few cases, discussed below, GRX1-roGFP2 was more responsive. In vitro, GRX1-roGFP2 is not oxidized by H_2_O_2_ while roGFP2-Orp1 is unresponsive to GSSG/GSH but, in the presence of GSH/GSSG, GRX1-roGFP2 is oxidized by H_2_O_2_ (Gutscher et al., 2008, 2009). Therefore, the close coupling of probe redox state in vivo most likely reflects oxidation of the thiol pool when H_2_O_2_ increases. This generally coupled response of probe oxidation was also observed during high light and methyl viologen-induced oxidative stress (Ugalde et al., 2021). However, it is critical to note that the redox state of both probes is also dependent on the capacity to reduce them via the thiol system which could be influenced during PTI and could differ between subcellular compartments. We find that the initial oxidation in the cytosol previously measured by roGFP2-Orp1 oxidation in response to flg22 (Nietzel et al., 2019) is followed several hours later by a much stronger transient oxidation. Critically, as discussed below, this response is much less pronounced in organelles and is independent of the apoplastic oxidative burst mediated by the RBOHD isoform of NADPH oxidase. The PTI responses are summarized diagrammatically in Figure 7.

### NADPH oxidases and peroxidases are not fully required for PAMP-induced oxidation of cytosolic roGFP2-Orp1

It was previously noted that flg22 caused a rapid luminol-measured burst followed by a delayed oxidation of cytosolic roGFP2-Orp1 but the relationship between these events was not resolved (Nietzel et al., 2019). The use of mutants in apoplastic ROS production and protein kinases BIK1 and BAK1 involved in PAMP perception has provided additional information. As expected (Zhang et al., 2007), the flg22-induced apoplastic ROS burst measured by luminol was rapid and transient and strictly dependent on RBOHD but not RBOHF. Surprisingly, the subsequent oxidation of cytosolic roGFP2-Orp1 was independent of both RBOH isoforms but notably, in the *rbohD* mutant, there was a delay in cytosolic roGFP2-Orp1 oxidation, suggesting that part of the cytosolic oxidation is dependent on H_2_O_2_ produced in the initial RBOHD-dependent burst. It is possible that other RBOH isoforms contribute to cytosolic roGFP2-Orp1 oxidation, but this is unlikely since DPI decreased the initial probe oxidation but did not affect it in the longer term. Therefore, it is apparent that PAMP treatment induces oxidation of roGFP2-Orp1 in an NADPH oxidase-independent manner. Activation of other apoplastic ROS producing enzymes, such as type III peroxidases is another possibility since they have been reported to be involved in the PAMP-induced ROS production (Bindschedler et al., 2006; Daudi et al., 2012). Analysis of the four peroxidase mutants suggested that these isoforms do not play major roles in PAMP-induced roGFP2-Orp1 oxidation although PRX34 may contribute to sustained roGFP2-Orp1 oxidation in the long term and *PRX4* up-regulation boosted PAMP-triggered H_2_O_2_ production in the cytosol. Functional redundancy is likely to occur as PRXs belong to a large multigenic family of 73 members (Valerio et al., 2004). Our results suggest that NADPH oxidases and apoplastic peroxidases may act additively. It should be noted that the fluorescence signals from the biosensors will mostly derive from the epidermal pavement cells, guard cells, and palisade cells, leaving the possibility that the various oxidative events occur in different cell types but is likely to be dominated by the palisade cells.

### Two phases of ROS production in the apoplast and of roGFP2-Orp1/GRX1-roGFP2 oxidation in the cytosol

Consistent with recent results (Ngou et al., 2021; Yuan et al., 2021), we observed a second flg22-triggered ROS burst in the apoplast less intense than the first apoplastic ROS burst but which lasted longer from 2 to 8 h. Compared to the apoplast, the second PAMP-triggered roGFP2-Orp1/GRX1-roGFP2 oxidation event in the cytosol and nucleus started later at 4 h, was about six-fold more pronounced in *R*/*R* value than the first increase in oxidation and lasted for 8 h. Importantly, the second phase of oxidation in the cytosol was not affected in *rbohD*, *rbohF*, *prx33-3*, and *prx34-2* mutants but was partially reduced in *bak1-5* and *bik1* mutants. Therefore, PAMP signaling through these kinases activates cytosolic oxidation independently of NADPH oxidase mediated H_2_O_2_ production. Interestingly, the second phase of oxidation was reduced after *Pst* DC3000 inoculation compared to the *Pst hrpA* strain defective in effector delivery. It is notable that a transcriptome time course comparing *Pst* DC3000 and *Pst hrpA* shows that effector-driven gene expression peaks at ∼6 h post-inoculation (Lewis et al., 2015), providing the possibility that metabolism could be affected during the second oxidation event. This is the time at which effector-driven decrease in photosynthesis occurs (de Torres Zabala et al., 2015). By contrast, a similar extent of GRX1-roGFP2 oxidation was observed after infection with WT *Pst* DC3000 or *Pst hrpA*. An increase in E_GSH_ in the cytosol was also observed in tobacco (*Nicotiana tabacum*) 6 h after infection with virulent *Pseudomonas* bacteria but the comparison with T3S-deficient bacteria or a PAMP was not performed (Matern et al., 2015). Our results suggest that bacterial effectors decrease roGFP2-Orp1 oxidation in the cytosol and organelles. Similarly, oxidation of a fluorescein-based ROS probe occurred in the cytosol and chloroplasts ∼4 h after inoculation with *Pst hrpA* but was suppressed with *Pst* DC3000 (de Torres Zabala et al., 2015). In this case, chloroplast oxidation was attributed to photosynthetically produced ROS but, it is important to note the current experiments were done in the dark, so photosynthesis is not involved. However, together, the results suggest bacterial effectors may reduce intracellular H_2_O_2_ production as a strategy to impair defense responses. Although roGFP2-Orp1 and GRX1-roGFP2 are more oxidized in chloroplasts, mitochondria, and peroxisomes than in the cytosol in unstressed conditions, organelles may have a better capacity to overcome excess ROS (due to activity of antioxidant enzymes) during PTI which might otherwise induce programmed cell death (Camejo et al., 2016).

### *rbohF* and *vtc2-4* are sensitive to roGFP2-Orp1 oxidation caused by H_2_O_2_ and flg22

The results indicate that RBOHF is important for maintaining the function of the antioxidant system. Unlike *rbohD,* the *rbohF* mutant has a small increase in the oxidation state of cytosolic/nuclear roGFP2-Orp1 compared to Col-0, and importantly, when exposed to H_2_O_2_, probe oxidation is greater than in Col-0. This conclusion is supported by decreased rosette size and increased bleaching of older leaves seen in *rbohFcat2* double mutants (Chaouch et al., 2012), suggesting that *rbohF* mutant has a decreased capacity to remove the excess H_2_O_2_ in the *cat2* catalase mutant. Interestingly, the dichlorofluorescein ROS-sensitive dye used by Chaouch et al. (2012) was not able to detect differences in between Col-0 and *rbohF* as compared to our measurements with roGFP2-Orp1. These results are consistent with a less active antioxidant system and, indeed, *rbohF* has lower expression of cytosolic ascorbate peroxidase (APX1), an enzyme known to be important in H_2_O_2_ removal (Chaouch et al., 2012). Related to this observation, we find that the ascorbate biosynthesis mutant *vtc2-4* (with ∼20% wild-type ascorbate, (Lim et al., 2016)) is also more susceptible to oxidation in response to H_2_O_2_ and flg22. The small stature and stress sensitivity of *rbohF* suggests a role for ROS production by RBOHF in various aspects of plant growth and development (Chaouch et al., 2012) and control over the antioxidant system.

### What is the cause of NADPH oxidase/peroxidase-independent cytosolic oxidation during PTI?

As discussed previously, the initial cytosolic oxidation and the large second oxidation of roGFP2-Orp1 biosensor are essentially independent of NADPH oxidases and apoplastic peroxidases and follow just after the RBOHD-dependent apoplastic oxidative bursts (Figure 7). The cause of cytosolic oxidation is not known and will require further investigation. If it is caused by increased H_2_O_2_ production, then the source would need to be identified. It is of note that peroxisomal GRX1-roGFP2 was more oxidized than roGFP2-Orp1 after bacteria treatments, and this response was noted previously in seedlings treated with flg22 (Bratt et al., 2016). Peroxisomes contain H_2_O_2_-producing oxidases and have proposed roles in localized response to pathogens (Koh et al., 2005), but lack of roGFP2-Orp1 oxidation suggests that they are not acting as a strong source of H_2_O_2_ during PTI. Peroxisomal GRX1-roGFP2 oxidation could indicate a lack of capacity to reduce the probe in this compartment. Cytosolic H_2_O_2_ sources are less obvious but might include autoxidation of flavin containing enzymes which are likely the main cytosolic ROS source in bacteria (Imlay, 2008; Smirnoff and Arnaud, 2019). The principal H_2_O_2_ removers are catalase (in peroxisomes), peroxiredoxins, glutathione peroxidase-like enzymes, and ascorbate peroxidases (Smirnoff and Arnaud, 2019) so that inhibition of their activity could decrease the scavenging of H_2_O_2_ produced by background metabolism or apoplast. We found that the low ascorbate *vtc2-4* mutant accumulates more H_2_O_2_ in response to flg22, most likely because of compromised H_2_O_2_ removal by ascorbate peroxidase, suggesting that inactivation of antioxidant defenses would be sufficient to increase H_2_O_2_. However, APX and CAT activities were not affected by flg22 treatment (Supplemental Figure S6). It is also possible that re-reduction of the biosensors through glutaredoxins and thioredoxins is inhibited during PTI. The observed decrease in expression of a number of genes coding for antioxidant enzymes in the chloroplast (SOD, MDAR, DHAR, GPX, PrxR, and Trx) or in the cytosol (Trx and GRX) upon PAMP perception or bacterial infection (Supplemental Figure S5) may explain the increase in roGFP2-Orp1 oxidation in these compartments. However, because these genes belong to multigenic families, further research is required to identify which ones are important for H_2_O_2_ scavenging and signaling during plant immunity. BAK1 was essential for PAMP-dependent biosensor oxidation, while *bik1* was partially defective. These kinases, possibly along with associated mitogen activated protein kinase cascades or calcium-dependent protein kinases (CPKs), could therefore phosphorylate target proteins to activate H_2_O_2_ production, decrease H_2_O_2_ scavenging or decrease the capacity of the systems that reduce the biosensors although there are no obvious examples. Interactions with nitric oxide are possible. It is interesting to note that the NO donor SNP oxidized both the probes. Increased NO production occurs during PTI (Yu et al., 2017) and is associated with protein *S*-nitrosylation (Begara-Morales et al., 2016; Lawrence et al., 2020; Bleau and Spoel, 2021). On the other hand, cPTIO had no effect on short-term biosensor oxidation suggesting that NO is at least not involved in the first oxidation event.

### Conclusion

The oxidative responses in response to PAMPs and *Pst* bacteria are summarized in Figure 7. We have characterized two apoplastic oxidative bursts dependent on the RBOHD isoform of NADPH oxidase. Using the H_2_O_2_ sensor roGFP2-Orp1 and the GSH redox sensor GRX1-roGFP2, targeted to various subcellular compartments, an NADPH oxidase-independent cytosolic oxidation occurs in two phases: a small oxidation following the first apoplastic burst and a large oxidation following the second apoplastic burst. The cause and function of these cytosolic oxidation events require further investigation. It is tempting to speculate that the RBOHD-mediated apoplastic burst is required for systemic signaling via the ROS wave (Fichman et al., 2019) as well as for local responses, while cytosolic oxidation is related to local defense.

## Materials and methods

### Plant materials and growth conditions

The cytosolic/nuclear roGFP2-Orp1 line (Nietzel et al., 2019), cytosolic/nuclear GRX1-roGFP2 (Marty et al., 2009), mitochondrial roGFP2-GRX1 (Albrecht et al., 2014), peroxisomal GRX1-roGFP2 (Rosenwasser et al., 2011), chloroplastic GRX1-roGFP2 lines (Park et al., 2013), and all mutant lines are in Arabidopsis (*Arabidopsis thaliana*) Col-0 background. *bik1* (SALK_005291), *rbohD* (CS9555), *rbohF* (CS9557), *prx4-2* (SALK_04473°C), *prx33-3* (GK-014E05), *prx34-2* (GK-728F08), *prx71-1* (SALK_123643C), and *vtc2-4* (SAIL_769_H05) were previously described (Torres et al., 2002; Veronese et al., 2006; Lim et al., 2016; Arnaud et al., 2017). The *bak1-5* mutant was genotyped according to (Schwessinger et al., 2011). All T-DNA insertion mutants were confirmed by polymerase chain reaction (PCR) genotyping prior to use (Supplemental Table S1). F3 homozygous plants of the double transgenic lines made with roGFP2-Orp1 and the above mutants were generated by crossing homozygous parental lines. The progeny was selected on appropriate antibiotics and genotyping by PCR. Four- to five-week-old plants grown on soil in a growth chamber under short-day conditions (10 h light at 22°C/14 h dark at 19°C), at 60% humidity and illuminated with fluorescent tubes at 100 µmol m^−2^ s^−1^ light intensity were used for all the experiments.

### Plasmid constructions and generation of transgenic plants

All constructs were generated and assembled by GoldenGate cloning (Weber et al., 2011; Engler et al., 2014) using Bsa1 and Bpil restriction enzymes in the modules described in Supplemental Table S1. The full-length coding sequence (CDS) of roGFP2-Orp1 was amplified from genomic DNA of Arabidopsis Col-0 expressing roGFP2-Orp1 (Nietzel et al., 2019) with primers roGFP2-Orp1-F and roGFP2-Orp1-R listed in Supplemental Table S1. The peroxisomal targeting sequence serine-lysine-leucine (SKL) was introduced at the C-terminus of roGFP2-Orp1 using the primer roGFP2-Orp1-SKL-R. The hygromycin CDS was amplified from the plasmid pCAMBIA1302 with the primers Hygro-F and Hygro-R (Supplemental Table S1). roGFP2-Orp1, roGFP2-Orp1-SKL, and hygromycin CDS were subcloned in pICH41308 (Weber et al., 2011). Nuclear localization signal (NLS) derived from Simian Virus 40 (Kalderon et al., 1984) was obtained by annealing two oligonucleotides NLS-F and NLS-R (Supplemental Table S1), encoding the amino acid residues MLQPKKKRKVGG. The nuclear export signal (NES) from the protein kinase inhibitor (Wen et al., 1995) was generated by annealing the NES-F and NES-R oligonucleotides encoding the amino acid residues MLQNELALKLAGLDINKTGG. NLS and NES targeting sequences were subcloned in pAGM1276 (Weber et al., 2011). The modules pICH78133, pAGM1482, and pICH78141 (Engler et al., 2014) were used for targeting roGFP2-Orp1 in chloroplasts, mitochondria, and apoplast, respectively. The different targeting sequences used for cloning are described in Supplemental Table S1. The final constructs for expressing roGFP2-Orp1 in nuclei, cytosol, chloroplasts, mitochondria, peroxisomes, and apoplast were assembled in GoldenGate reactions from modules listed in Supplemental Table S1. The fidelity of all constructs was confirmed by sequencing.

Arabidopsis Col-0 plants were transformed using the GV3101 strain of *Agrobacterium tumefaciens* according to the floral dip protocol (Clough and Bent, 1998). Transgenic lines were isolated on plates containing 1/2 MS (Sigma), MES-KOH (Sigma), pH 5.7, and 0.8% (w/v) agar (Neogen) supplemented with 20 µg mL^−1^ hygromycin B (Invitrogen). Transgenic lines were screened for 3:1 segregation of the resistance marker and fluorescence intensities of the respective sensors and raised to homozygous T3 lines. For each construct at least 10 independent lines were screened and 2 lines showing strong expression based on fluorescence intensity were selected. The expression of roGFP2-Orp1 was observed throughout the plant development from seedlings (root and shoot) to adult plants. Random silencing of the transgene could be observed in 4-week-old adult plants.

### Chemicals

Purified chemicals, except the flg22 and elf18 peptides (Peptron, Korea), were purchased from Sigma. Control solutions were 10 mM MES-KOH pH 6.15, 30 mM KCl buffer containing 1% ethanol for 1 mM 2-Phenyl-4,4,5,5-tetramethylimidazoline-1-oxyl 3-oxide (cPTIO), 0.1% dimethyl sulfoxide for 20 µM DPI, and water for 10 µM to 100 mM hydrogen peroxide (H_2_O_2_), 50 mM 1,4-DTT, 50 µM to 50 mM SNP, 10 nM to 10 µM flg22 or elf18.

### Bacterial strains and preparation

The WT bacterial strain *Pst* DC3000 and the T3S-deficient *Pst* DC3000 *hrpA* mutant strain (de Torres Zabala et al., 2015) were cultivated overnight at 28°C in King's B medium supplemented with Kanamycin and Rifampicin (each at 100 µg mL^−1^). Bacteria were collected by centrifugation at 3000 *g* for 5 min at room temperature and washed twice in 10 mM MgCl_2_. Leaf discs were inoculated with a bacterial solution of 10^8^ cfu mL^−1^ in 10 mM MgCl_2_.

### Multiwell plate reader-based fluorimetry

Because of random silencing of the transgene after 3 weeks of growth, plants expressing roGFP2-Orp1 were first selected with an epifluorescence binocular microscope. Leaf discs (6 mm diameter) were placed in a 96-well plate, immersed in 200 µL 10 mM MES-KOH pH 6.15, 30 mM KCl with their abaxial side facing up, and incubated for 2 h at 21°C under laboratory lighting (PPFD ∼ 10 µmol m^−2^ s^−1^) for recovery after wounding. roGFP2-Orp1 was excited sequentially at 400 ± 8 nm and 485 ± 8 nm in a CLARIOstar plate reader (BMG Labtech) and emission was recorded at 525 ± 20 nm with a gain set at 2,000 and 1,500 for the 400 and 485 nm excitations respectively. Each leaf disc was scanned from the top with the fluorescence recorded and averaged from 76 flashes per well organized as a spiral of 5 mm diameter. The initial 400/485 ratio of the resting state of leaf discs was estimated by reading the wells at 5 min intervals for 15 min before treatment. For each treatment, the emission of six Col-0 (WT) leaf discs was averaged and subtracted for all the data points to correct for background fluorescence. The degree of probe oxidation and the DR were calculated according to Schwarzländer et al. (2008). Changes in fluorescence over time were expressed relative to the initial ratio Ri as *R*/*R*_i_ to allow for differences in resting 400/485 ratio between leaf discs. Chemicals or control solutions were simply added to the wells. For bacterial inoculations and long-term PAMP treatments, leaf discs were first vacuum infiltrated for 30 min before adding bacteria, PAMPs, or mock solution (10 mM MgCl_2_). However, the vacuum treatment induced an overall decrease in fluorescence so that the nuclear-targeted roGFP2-Orp1 fluorescence became lower than background fluorescence overtime and was excluded from analyses.

### Luminol assay

Leaf discs of 6 mm diameter were cut into four equal pieces, immersed in distilled water, and incubated for 3 h minimum at room temperature for recovery after wounding. Before starting the assay, water was exchanged by a solution containing 100 µM luminol and 10 µg mL^−1^ horseradish peroxidase (≥250 units mg^−1^ solid). After adding control solution or 1 µM flg22, the luminescence was measured immediately using a CLARIOstar plate reader (BMG Labtech) with a reading time of 2 s.

### Microscopic analysis

Epidermal peels from rosette leaves were immersed in 10 mM MES-KOH pH 6.15, 30 mM KCl, incubated for 2 h in the growth chamber at 22°C (PPFD ∼ 100 µmol m^−2^ s^−1^) for recovery after wounding. Epidermal peels were mounted under a Leica DM2500 microscope. Images were collected with a 40X lens (Plan-Apochromat, 0.8 numerical aperture). roGFP2-Orp1 and chlorophyll fluorescence emissions were detected through a long-pass filter with a cutoff wavelength at 515 nm after excitation at 470 ± 20 nm. The fluorescence emission of roGFP2-Orp1 was also detected through a band-pass filter at 525 ± 50 nm following excitation at 470 ± 40 nm.

### Ascorbate peroxidase and catalase enzyme assays

Leaf discs were prepared and treated with control solution or 1 µM flg22 for 2 h in the same way as for the roGFP2-Orp1 fluorimetry method. Enzymes were extracted according to (Colville and Smirnoff, 2008) with some modifications. Leaf discs (20 mg fresh weight) were homogenized in 200 µL ice-cold extraction buffer [50 mM potassium phosphate (pH 7.0), 1 mM Na_2_EDTA, 20% (v/v) glycerol, 0.1% (v/v) Triton X-100, and 2 mM DTT and 1 mM ascorbate, which were added just before use], centrifuged at 16,000 *g* for 10 min at 4°C, and kept on ice until assayed. The APX assay consisted of 237.5 µL of 50 mM potassium phosphate buffer (pH 7.0) containing 1 mM Na_2_EDTA, 2.5 µL of 25 mM ascorbate, 5 µL of extract, and 5 µL of 200 mM hydrogen peroxide in a total volume of 250 µL. The oxidation of ascorbate was followed at 280 nm (*ε* = 7.83 mM^−1^ cm^−1^). For catalase, the assay consisted of 240 µL of 50 mM potassium phosphate buffer (pH 7.0) containing 1 mM Na_2_EDTA, 5 µL of extract, and 5 µL of 2 M hydrogen peroxide in a total volume of 250 µL. The decomposition of H_2_O_2_ was followed at 240 nm (*ε* = 0.04 mM^−1^ cm^−1^).

### Analysis of transcriptome databases

TAIR accession numbers of genes coding for antioxidant enzymes were retrieved from (https://itservices.cas.unt.edu/∼rmittler/genelist.htm) and their predicted subcellular localization was verified using the TAIR database (https://www.arabidopsis.org/). Heat maps of expression profile were generated using the iNID web interface (http://omics.sbmlab.com/inid/) with default parameters (Choi et al., 2014). The transcriptome data sets analyzed are time-course experiments on the leaves of 5-week-old Arabidopsis plants collected 2, 6, and 24 h after inoculation with the bacterial strains *Pst* DC3000 and *Pst* DC3000 *hrcC^−^* (ME00331), or 1 and 4 h after treatment with flg22 and lipopolysaccharide (LPS) elicitors (GSE5615). Genes were considered as differentially expressed with a *P*-value <0.05 and a log2 fold-change >0.58 (upregulated) or a log2 fold-change <−0.58 (down-regulated) for at least one-time point.

### Statistical analysis

The experiments reported here were repeated at least three times with similar results unless otherwise mentioned. Nontime course experiments were analyzed by Student's *t*-tests or ANOVAs followed by Tukey's honestly significant difference (HSD) post hoc test using R software (R Core Team, https://www.R-project.org). The time-course experiments, in which roGFP2-Orp1 and GRX1-roGFP oxidation state was followed after PAMP and H_2_O_2_ addition, were analyzed by two-way analysis of variance (ANOVA) using repeated measures for time. Significant differences between each treatment at each time were determined by Tukey's multiple comparisons test using GraphPad Prism v8 (GraphPad, San Diego, California, USA). All time-course effects discussed in the text are significant (*P* < 0.05) and the analysis is shown in Supplemental Tables S2 and S3.

### Accession numbers

The *A. thaliana* genes included in this study are as follows: *BAK1* (At4g33430); *BIK1* (At2g39660); *PRX4* (At1g14540); *PRX33* (At3g49110); *PRX34* (At3g49120); *PRX71* (At5g64120); *RDOHD* (At5g47910); *RBOHF* (At1g64060); and *VTC2* (At4g26850).

## Supplemental data

The following materials are available in the online version of this article.

**Supplemental Figure S1.** Constructs, phenotypes, and localization of roGFP2-Orp1 targeted to different subcellular compartments.

**Supplemental Figure S2.** In vivo characterization of cytosolic/nuclear roGFP2-Orp1 oxidation in response to H_2_O_2_ and flg22.

**Supplemental Figure S3.** elf18-triggered roGFP2-Orp1 oxidation in mutants of PTI regulators, NADPH oxidases, and apoplastic peroxidases.

**Supplemental Figure S4.** In vivo characterization of roGFP2-Orp1 and GRX1-roGFP2 responses to the NO scavenger cPTIO and the NO donor SNP.

**Supplemental Figure S5.** The expression of antioxidant genes is deregulated by bacteria or PAMPs in leaves.

**Supplemental Figure S6.** Ascorbate peroxidase and catalase activities are not affected by flg22 in leaves of *rbohD* and *bak1* mutants.

**Supplemental Table S1.** PCR primers for genotyping and GoldenGate cloning, transit peptides, and GoldenGate parts.

**Supplemental Table S2.** Statistical analyses for data are shown in Figures 1–6.

**Supplemental Table S3.** Statistical analyses for Supplemental Data.

## Supplementary Material

kiac603_Supplementary_Data
