# Supplementary material for: Organelle-targeted biosensors reveal distinct oxidative events during pattern-triggered immune responses
Source: Plant Physiol. 2022 Dec 30;191(4):2551–69. doi: 10.1093/plphys/kiac603 (PMC10069903; doi:10.1093/plphys/kiac603)
Supplement: kiac603_Supplementary_Data [file kiac603_supplementary_data.zip › Supplemental Figs 1 to 6 and Supplemental Table 1 Higher res please replace.pdf]

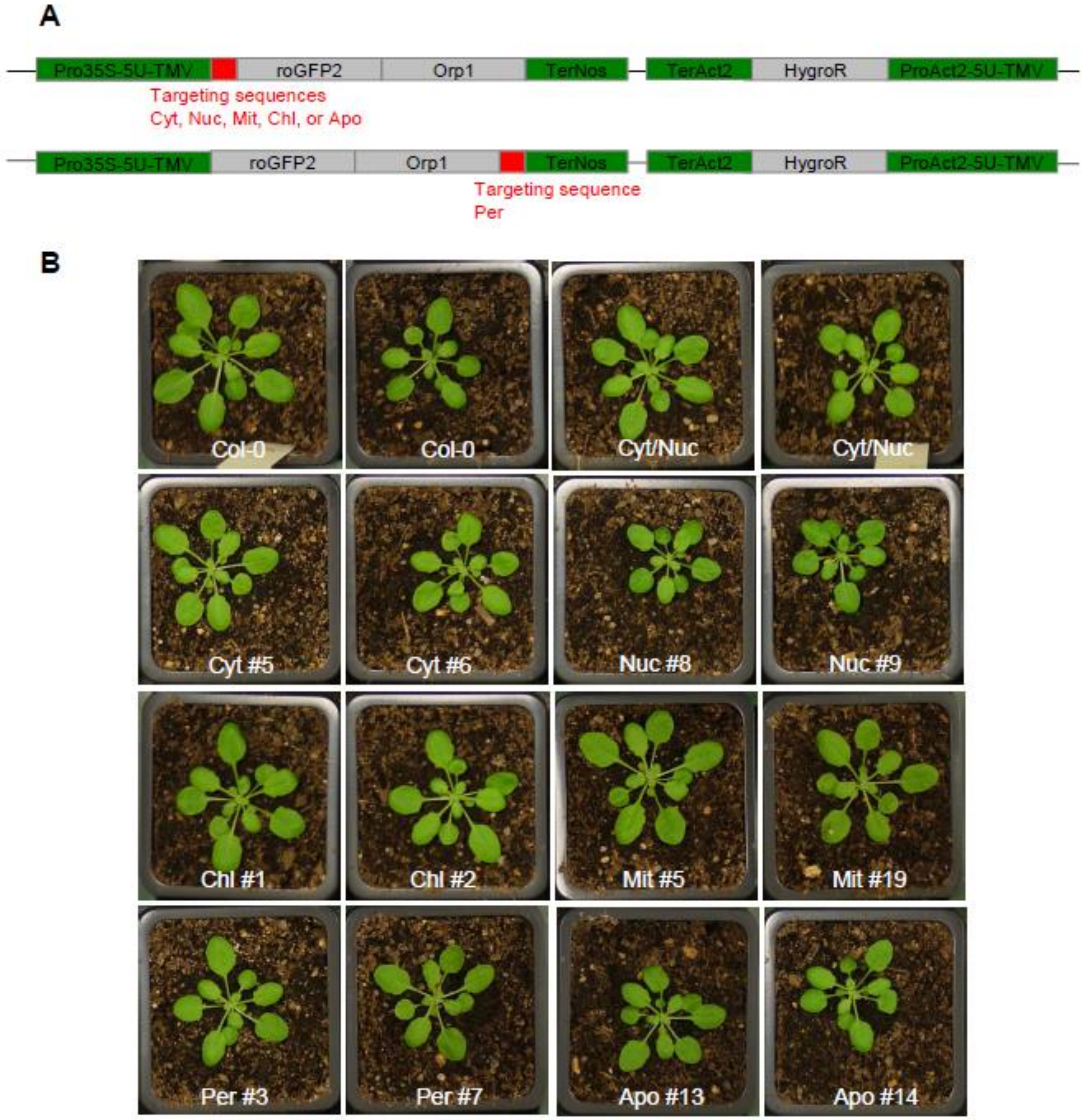

Supplemental Figure S1

A, Schematic representation of the constructs used to target roGFP2-Orp1 in the cytosol (Cyt), nuclei (Nu), chloroplasts (Chl), mitochondria (Mit), peroxisomes (Per) or apoplast (Apo). Pro35S, promoter 35S (Cauliflower Mosaic Virus); 5U-TMV, 5'UTR Ω (Tobacco Mosaic Virus); ProAct2, promoter Actin2 (AT3G18780, *A. thaliana*); TerNos, Nos (*A. tumefaciens*) 3'UTR and terminator; TerAct2, Actin2 (*A. thaliana*) 3'UTR and terminator. **B**, Phenotype of representative 4-week-old Arabidopsis Col-0 wild-type plants and Arabidopsis lines overexpressing roGFP2-Orp1 in the cytosol and nuclei (Cyt/Nu), cytosol (Cyt), nuclei (Nu), chloroplasts (Chl), mitochondria (Mit), peroxisomes (Per) or apoplast (Apo).

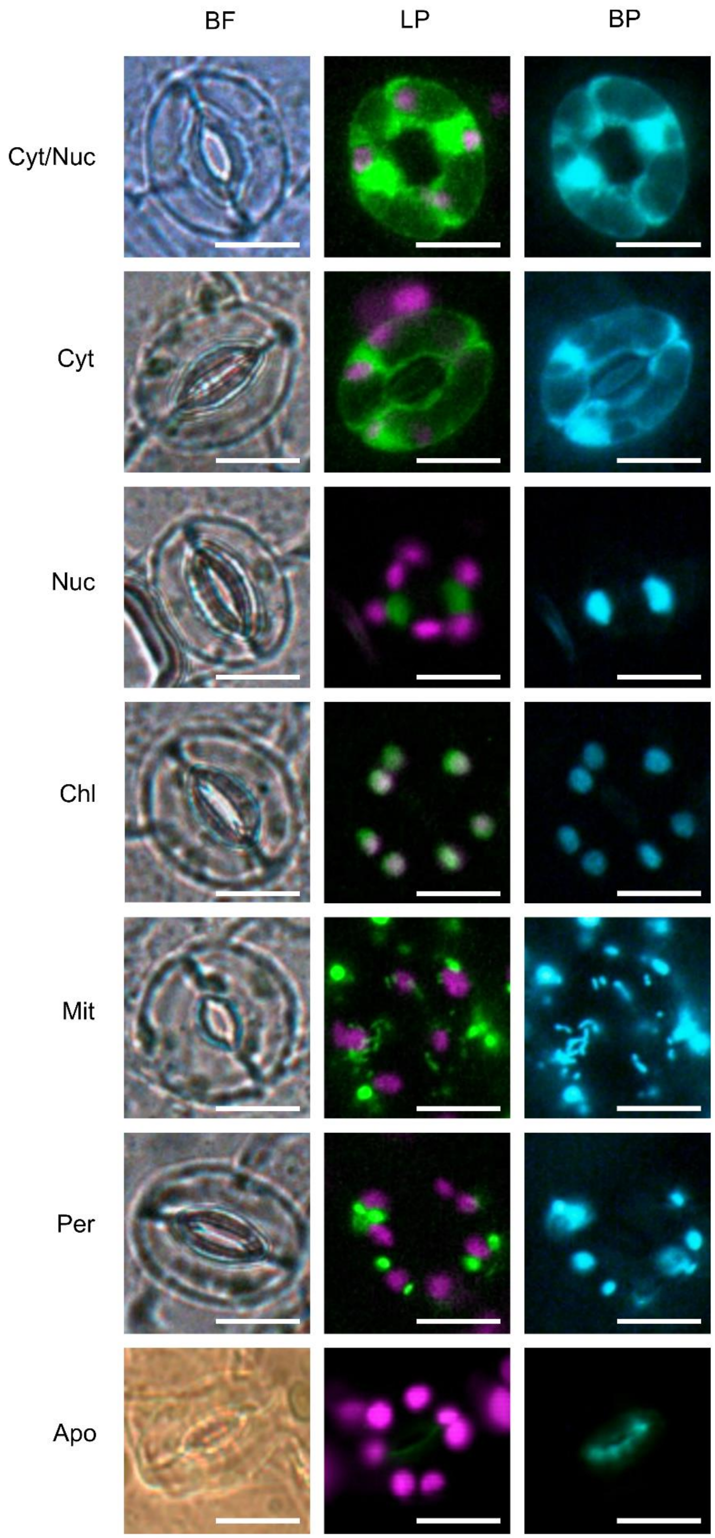

Supplemental Figure S1

C. Localisation of roGFP2-Orp1 in different subcellular compartments. The LP fluorescence images are already shown in Fig. 1A and this figure shows additional bright field (BF) and bandpass (BP) images. Representative images of roGFP2-Orp1 targeted to the cytosol (Cyt), nuclei (Nuc), chloroplasts (Chl), mitochondria (Mit), peroxisomes (Per) and apoplast (Apo) in guard cells from leaf epidermal strips of 5-week-old plants. Long-pass images represent the fluorescence emission through a long-pass filter with a cut-on wavelength at 515 nm after excitation at 470 ± 20 nm. roGFP2-Orp1 and chloroplast fluorescence are depicted in green and magenta respectively. Band-pass images represent the fluorescence emission at 525 ± 50 nm following excitation at 470 ± 40 nm. roGFP2-Orp1 fluorescence is depicted in cyan. Scale bars represent 10 µm.

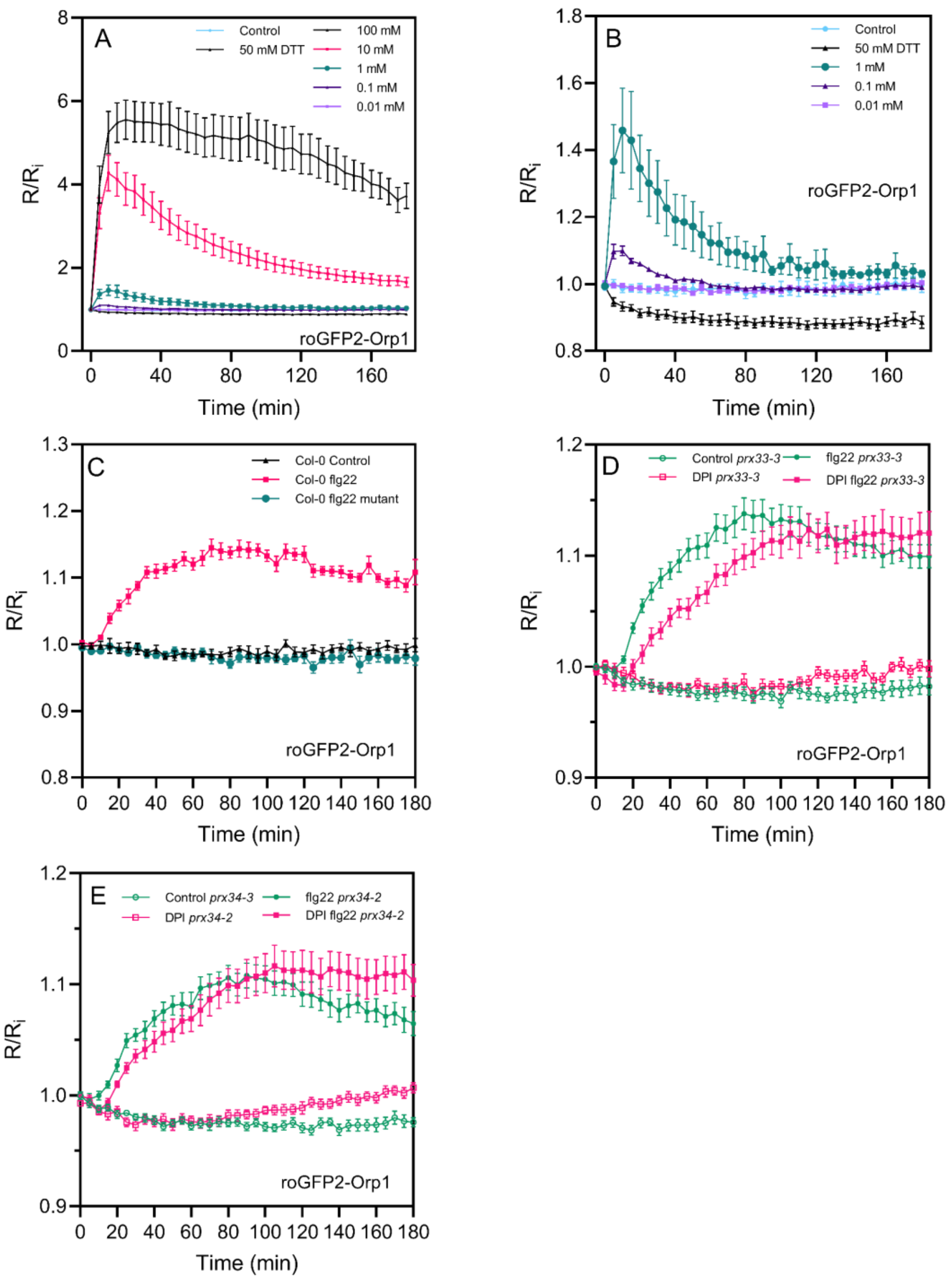

Supplemental Figure S2 *In vivo* characterisation of cytosolic/nuclear roGFP2-Orp1 oxidation in response to H_2O_2 and flg22.

A-B, *In vivo* characterisation of cytosolic/nuclear roGFP2-Orp1 oxidation and reduction kinetics in response to H_2O_2 and DTT. Leaf discs from rosette leaves of 5 week-old plants were exposed at t = 0 min to control solution, various concentrations of H_2O_2, or 50 mM DTT. For clarity high concentrations of H_2O_2 are shown in (**A**) and lower doses are shown in (**B**).

C, Kinetics of cytosolic/nuclear roGFP2-Orp1 oxidation in leaves of Col-0 WT in response to flg22 from *Agrobacterium tumefaciens*. Leaf discs were exposed at t = 0 min to control solution, 1 µM flg22 or 1 µM mutated flg22 (flg22 mutant).

D-E, Effect of the NADPH oxidase inhibitor DPI on flg22-induced oxidation of roGFP2-Orp1 in *prx33-3* (**D**) and *prx34-2* (**E**) mutants c to Col-0 WT. After 2 hours of pre-treatment with Control solution or 20 µM DPI, leaf discs were exposed at t = 0 min to Control solution or 1 µM flg22. In (A-E), the 400/485 nm fluorescence ratio (R) was measured over time by multiwell fluorimetry (excitation at 400 ± 8 and 485 ± 8 nm; emission, 525 ± 20 nm) and expressed relative to the mean initial ratio (Ri) before treatment (R/Ri). In (A-C), a representative experiment is shown ($n \geq 5$) and in (D-E), data are means ± SE of three independent experiments ($n \geq 10$). 2-way ANOVA using repeated measures for time and Tukey's multiple comparisons analyses are shown in Supplemental Table S3. The 400/485 nm fluorescence ratios for fully reduced/fully oxidised probes in the cytosol (Fig. 1) were 0.89/5.08.

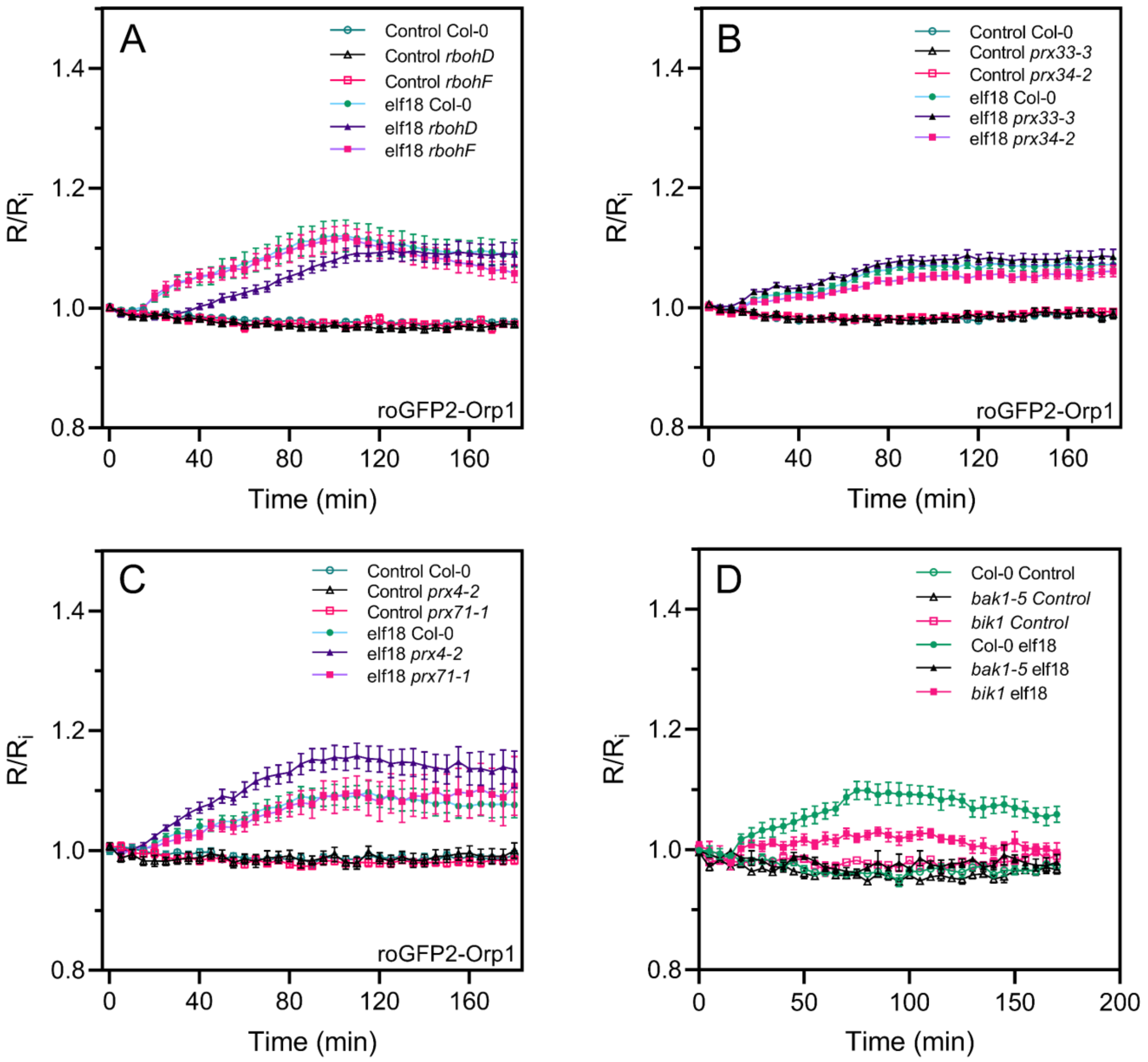

Supplemental Figure S3 elf18-triggered roGFP2-Orp1 oxidation in mutants of PTI regulators, NADPH oxidases and apoplastic peroxidases.

A-**D**, Kinetics of cytosolic/nuclear roGFP2-Orp1 oxidation in leaves of *rbohD* and *rbohF* (**A**), *prx33-3* and *prx34-2* (**B**), and *prx4-2* and *prx71-1* (**C**) and *bak1-5* and *bik1* (**D**) mutants in response to elf18. Leaf discs were exposed at t = 0 min to control solution or 1 µM elf18. The 400/485 nm fluorescence ratio (R) was measured over time by multiwell fluorimetry (excitation at 400 ± 8 and 485 ± 8 nm; emission, 525 ± 20 nm) and expressed relative to the mean initial ratio (Ri) before treatment (R/Ri). Data are means ± SE from at least three independent experiments (n ≥ 16, **B**-**D**). In (**A**), a representative experiment is shown (n ≥ 5). 2-way ANOVA using repeated measures for time and Tukey's multiple comparisons analyses are shown in Supplemental Table S3. The 400/485 nm fluorescence ratios for fully reduced/fully oxidised probes in the cytosol (Fig. 1) were 0.89/5.08.

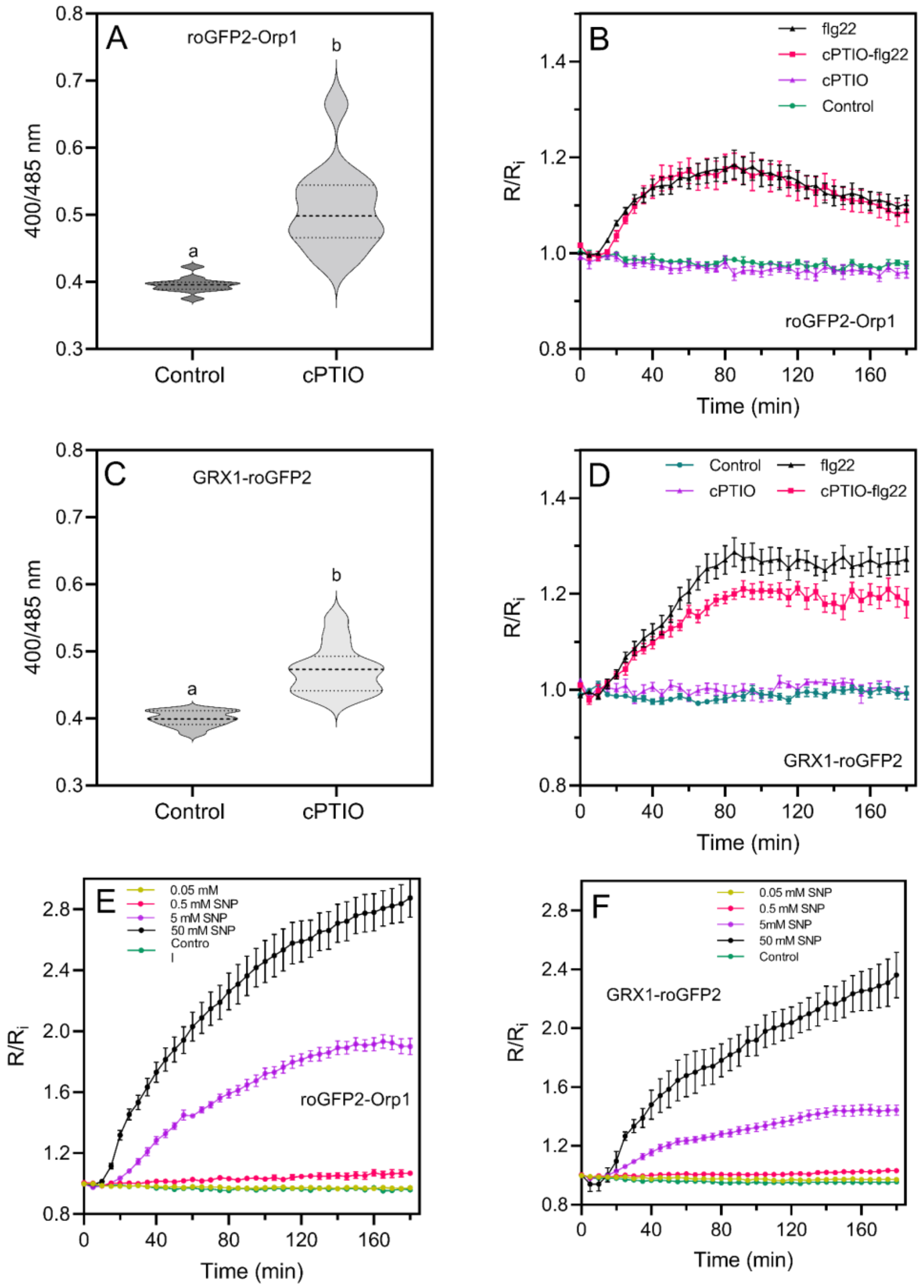

Supplemental Figure S4 *In vivo* characterisation of roGFP2-Orp1 and GRX1-roGFP2 responses to the NO scavenger cPTIO and the nitric oxide (NO) donor SNP.

A and **C**, Effect of the NO scavenger cPTIO on the oxidation state of roGFP2-Orp1 (**A**) and GRX1-roGFP2 (**C**). Leaf discs were treated for 2 hrs with 1 % ethanol as a Control or 1 mM cPTIO and the oxidation status of roGFP2-Orp1 and GRX1-roGFP2 (ratio 400/485 nm) was measured by multiwell fluorimetry. Data are means ± SE from a representative experiment (n = 12). Asterisks indicate statistically significant differences between Control and cPTIO treatments based on a two-tailed Student's *t*-test ($^{***}P$<0.001).

B and **D**, The response of roGFP2-Orp1 to the PAMP flg22 is not affected by the NO scavenger cPTIO. Effect of cPTIO on flg22-induced oxidation of roGFP2-Orp1 (**B**) and GRX1-roGFP2 (**D**). After 2 hrs of pre-treatment with Control solution or 1 mM cPTIO, leaf discs were exposed at t = 0 min to Control solution or 1 µM flg22.

E-F, Kinetics of roGFP2-Orp1 (**E**) and GRX1-roGFP2 (**F**) oxidation in leaves in response to NO. Leaf discs were exposed at t = 0 min to control solution or various concentration of the NO donor SNP. In (**B**, **D-F**), the 400/485 nm fluorescence ratio (R) was measured over time by multiwell fluorimetry and expressed relative to the mean initial ratio (Ri) before treatment (R/Ri). Data are means ± SE from a representative experiment (n = 6). 2-way ANOVA using repeated measures for time and Tukey's multiple comparisons analyses are shown in Supplemental Table S3. The experiments have been repeated at least twice with similar results.

The 400/485 nm fluorescence ratios for fully reduced/fully oxidised probes in the cytosol (Fig. 1) were 0.89/5.08.

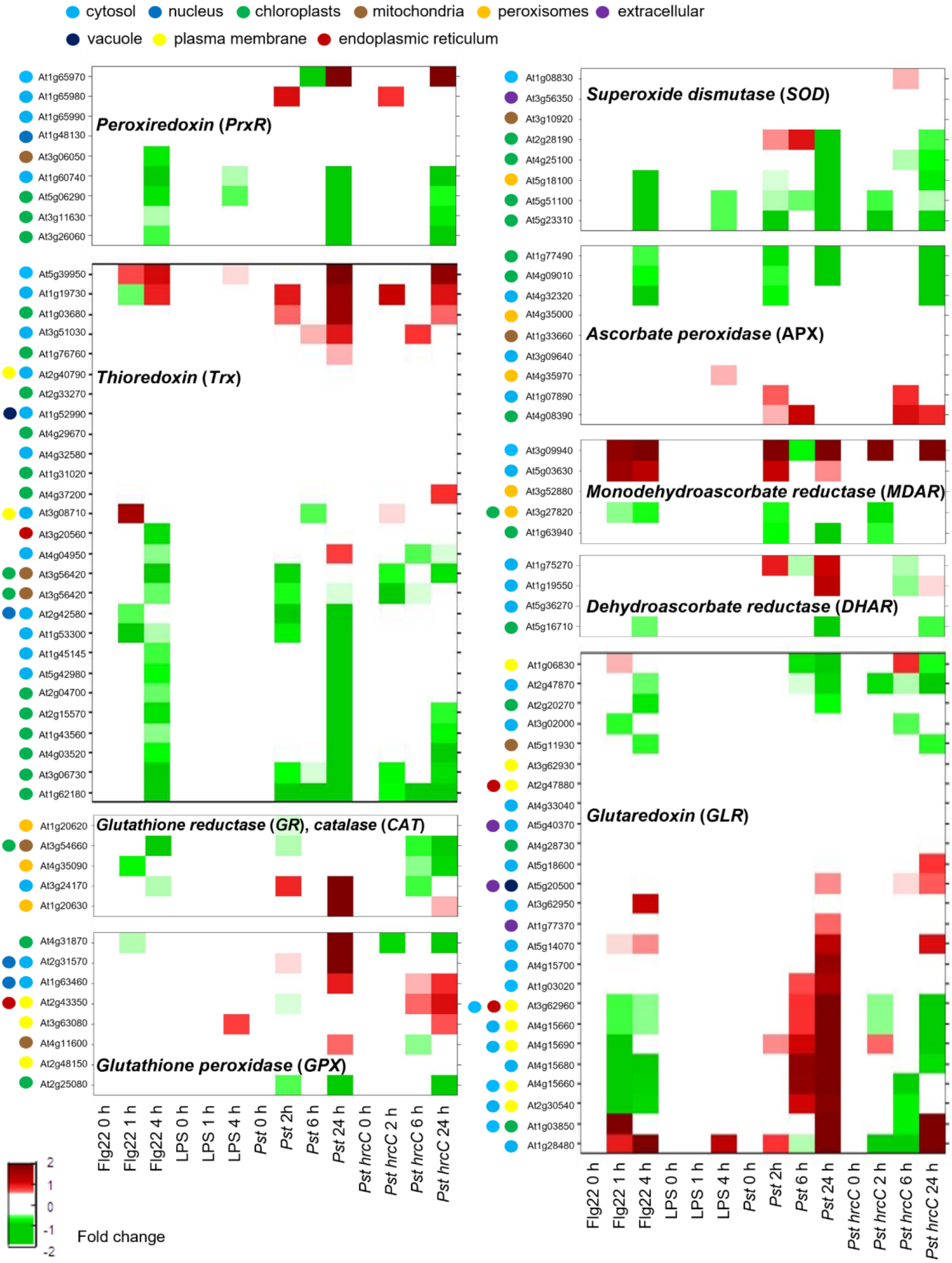

Supplemental Figure S5 The expression of antioxidant genes is deregulated by bacteria or PAMPs in leaves. Expression profile of *peroxiredoxin* (*PrxR*), *thioredoxin* (*Trx*), *glutathione reductase* (*GR*), *catalase* (*CAT*), *glutathione peroxidase* (*GPX*), *superoxide dismutase* (*SOD*), *ascorbate peroxidase* (*APX*), *monodehydroascorbate reductase* (*MDAR*), *dehydroascorbate reductase* (*DHAR*) and *glutaredoxin* (*GLR*) genes in response to elicitor treatments (flg22 and LPS), and infection with WT *Pst* DC3000 (*Pst*) and the disarmed *Pst hrcC* bacteria. The predicted subcellular localization of these antioxidant enzymes is indicated by different colors. Fold change (log2; treatment versus control, $P < 0.05$) are color-coded (red and green for relatively higher or lower expression, respectively) as indicated in the color bar. White indicates no change between conditions. Data are from GEO and AtGenExpress databases (GSE5615 and ME00331).

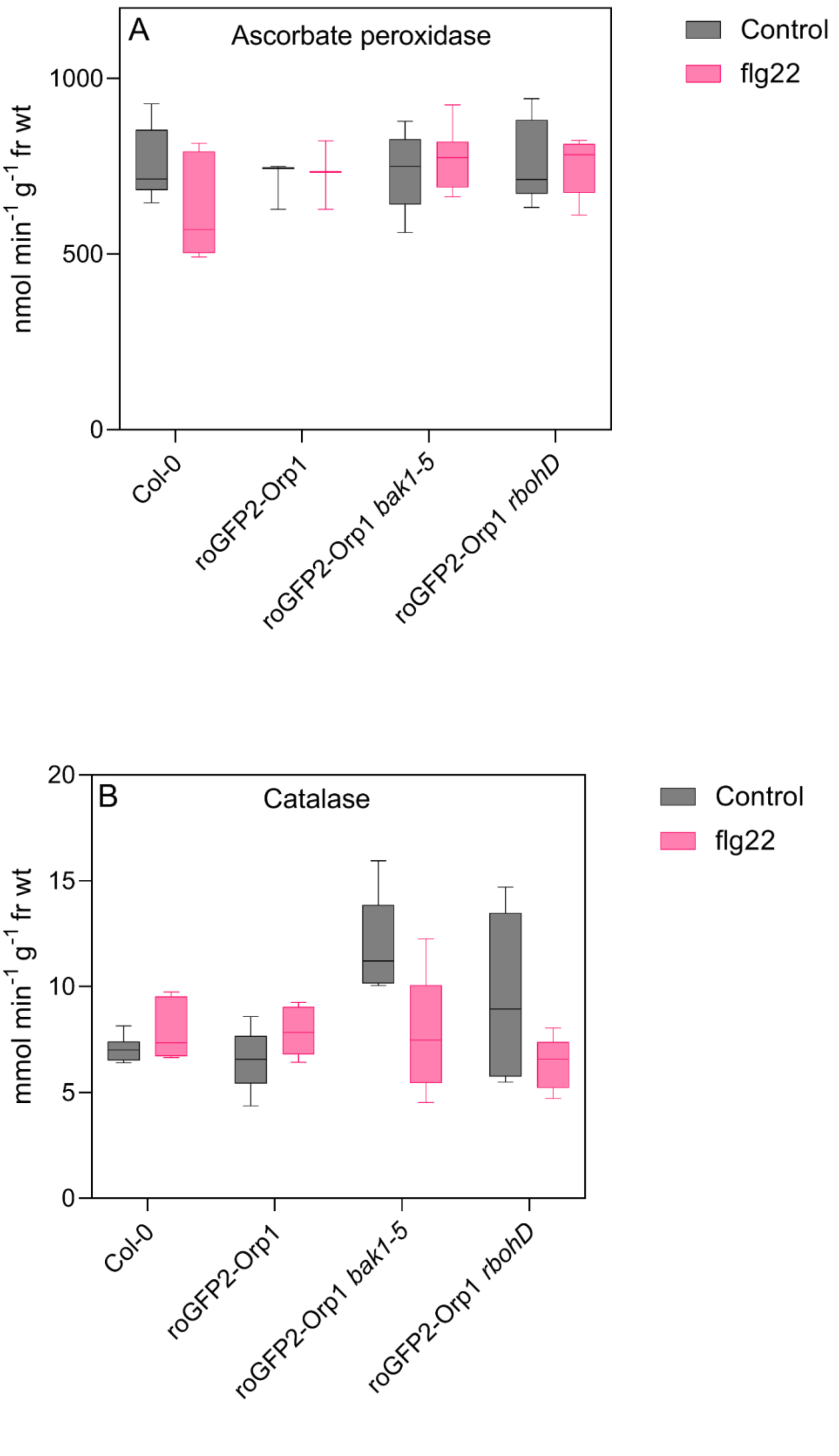

Supplemental Figure S6 Ascorbate peroxidase and catalase activities are not affected by flg22 in leaves of *rbohD* and *bak1* mutants. Activities of ascorbate peroxidase (A) and catalase (B) enzymes in Col-0 WT, roGFP2-Orp1 (cyt/nu), roGFP2-Orp1 *bak1-5*, and roGFP2-Orp1 *rbohD* mutants. Leaf discs from 5 week-old plants were treated for 2 hrs with control solution or 1 µM flg22. Bars represents median values boxes are 25th to 75th percentiles whiskers are max-min values. Data are from a representative experiment ($n \geq 3$). fr wt, fresh weight.

Supplemental Table S1

PCR primers used for genotyping and GoldenGate cloning.

Name	Forward primers 5' to 3'	Reverse primers 5' to 3'
**Genotyping**		
bak1-5 (dCAPS)	AAGAGGGCTTGCGTATTTACATGATCAGT	CGCGAGGCGAGCAAGATCAAAAG
bik1	CGATCCCGTCAAAGTGATATACC	CCGGATTGGTCTGACTCGGTTT
rbohD	CATGGGTTATTGCGTTTGTGTCGCCAA	GGATACTGATCATAGGCGTGGCTCCA
rbohF	ACTTCCGATATCCTTCAACCAACTCTTTG	CTCTCGTCGTTGATTTGTGACCAATACT
vtc2-4	AGCCTCCTGTAGCTTTTCTCGAG	GGCGAGAGCAGTAACCTCCTTA
prx4-2	GCGTTTAGGGCTATCGCAGAC	TCACCAGAACATCTCCACACGG
prx33-3	TGGAAATGCAAATTCAGCCCGA	CAGATCGAAATCCACTAAGACG
prx34-2	CACCCCTACCTTCTACGATAG	CCATTTGTTCCTCTGAAGCAAG
prx71-1	CGCCGTGAATGCTGGTTTCAG	CCCACGGCTTAGGTTGTTGTAG
Salk T-DNA	TGGTTCACGTAGTGGGCCATCG	
GABI-Kat T-DNA	ATATTGACCATCATACTCATTGC	
DsLox T-DNA	GCTCTTGCTAAGCTCCTCGAGTT	
SAIL-R	CCCAAATTACCAATACATTACACTAGC	
**GoldenGate cloning**		
roGFP2-Orp1-F	ttgaagacaaAATGGTGAGCAAGGGCGAG	
roGFP2-Orp1-R	ttgaagacaaaagcCTATTCCACCTCTTTCAAAAGTTC	
roGFP2-Orp1-SKL-R	ttgaagacaaaagcTTACAGCTTCGATCTTTCCACCTCTTTCAAAAGTTC	
NES-F	ttgaagacaaccATGCTGCAGAACGAGCTTGCTCTTAAGTTGGCTGGACTTGATATTAACAAGACTGGAGGAatgttgtcttcaa	
NES-R	ttgaagacaacatTCCTCCAGTCTTGTTAATATCAAGTCCAGCCAACTTAAGAGCAAGCTCGTTCTGCAGCATggttgtcttcaa	
NLS-F	ttgaagacaaccATGCTGCAGCCTAAGAAGAAGAGAAAGGTTGGAGGAatgttgtcttcaa	
NLS-R	ttgaagacaacatTCCTCCAACCTTTCTCTTCTTCTTAGGCTGCAGCATggttgtcttcaa	
Hygro-F	ttgaagacaaAATGAAAAAGCCTGAACTC	
Hygro-R	ttgaagacaaaagcCTATTTCTTTGCCCTCGGA	

Supplemental Table S1

Targeting peptides fused to the roGFP2-Orp1 or GRX1-roGFP2 lines used in this study

Lines	Organelles	Targeting sequences	References
GRX1-roGFP2 (Chl)	Chloroplasts	Transketolase target peptide (TKTP)	Park et al., 2013
roGFP2-GRX1 (Mit)	Mitochondria	Targeting peptide of Arabidopsis serine hydroxymethyltransferase (GenBank AJ271726.1)	Albrecht et al., 2014
GRX1-roGFP2 (Per)	Peroxisomes	SKL sequence	Rosenwasser et al., 2011
roGFP2-Orp1 (Nuc)	Nucleus	Nuclear localisation signal derived from Simian Virus 40 (NLS SV40)	Kalderon et al., 1984; this study
roGFP2-Orp1 (Cyt)	Cytoplasm	Nuclear Export Signal (NES) of the protein kinase inhibitor (PKI) from rabbit	Wei et al., 1995; this study
roGFP2-Orp1 (Chl)	Chloroplasts	Chloroplast transit peptide from the ribulose-1,5-bisphosphate carboxylase oxygenase (Rubisco) small subunit RbcS (a synthetic consensus of dicot sequences)	Marillonnet et al., 2004; Engler et al., 2014; this study
roGFP2-Orp1 (Mit)	Mitochondria	Targeting peptide derived from the subunit IV of cytochrome c oxidase (ScCoxIV) from *Saccharomyces cerevisiae*	Hurt et al., 1984; Engler et al., 2014; this study
roGFP2-Orp1 (Per)	Peroxisomes	SKL sequence for targetting to peroxisome	Sparkes et al., 2003; this study
roGFP2-Orp1 (Apo)	Apoplast	Signal peptide from the amylase OsRAmy3A from *Oryza sativa*	Engler et al., 2014; this study

Supplemental Table S1

Modules Golden Gate Plant Parts Kit

Annotation	Module Description	Vector name	Reference
tAct2	3'UTR, polyadenylation signal/terminator act2 (A. thaliana)	pICH44300	1
mCherry	CDS, mCherry variant of RFP (Discosoma sp.)	pICSL80007	1
p35S	promoter (1.3 kb), 35s (Cauliflower Mosaic Virus)	pICH41373	1
5U-TMV:Chl	5'UTR, Ω (Tobacco Mosaic Virus) + chloroplast transit peptide, RbcS (synthetic)	pICH78133	1
5U-TMV:Mito	(*Saccharomyces cerevisiae*)	pAGM1482	1
tNos	3'UTR, polyadenylation signal/terminator, nos (A. tumefaciens)	pICH41421	1
pNos-5U-TMV	promoter nos (A. tumefaciens)	pICH42211	1
p35S-5U-TMV	promoter (1.3 kb), 35s (Cauliflower Mosaic Virus) + 5'UTR Ω (Tobacco Mosaic	pICH51266	1
pAct2-5U-TMV	promoter, act2 (AT3G18780, A. thaliana) and 5'UTR, Ω (Tobacco Mosaic Virus)	pICH87644	1
5U-TMV:SP-OsRAmy3A	5'UTR, Ω (Tobacco Mosaic Virus) + signal peptide, RAmy3A (*Oryza sativa*)	pICH78141	1
tOcs	3'UTR, polyadenylation signal/terminator, ocs (*A. tumefaciens*)	pICH41432	1
5U-TMV	5'UTR, Ω (Tobacco Mosaic Virus)	pAGT707	1

Modules MoClo Plant Tool Kit

Annotation	Module Description	Vector Name	Reference
L0 NT1	Level zero acceptor for NT1 modules	pAGM1276	1
L0 CDS1	Level zero acceptor for CDS1 modules	pICH41308	1
L1 F1	Level 1 acceptor. Position 1. Forward orientation	pICH47732	1
L1 F3	Level 1 acceptor. Position 3. Forward orientation	pICH47751	1
L1 R2	Level 1 acceptor. Position 2. Reverse orientation	pICH47811	1
L2	Level 2 acceptor	pAGM4723	1
End-link 2	End-link 2 for assembling 2 level one part into a level 2 acceptor	pICH41744	1
End-link 3	End-link 3 for assembling 3 level one part into a level 2 acceptor	pICH41766	1

GoldenGate assembly level 0 (cloning of part of genes such as promoter, CDS, signal peptide...)

Annotation	Modules used	Reference
L0 Hygro	pICH41308, PCR product hygromycin	This study
L0 roGFP2-Orp1	pICH41308, PCR product roGFP2-Orp1	This study
L0 roGFP2-Orp1-SKL	pICH41308, PCR product roGFP2-Orp1-SKL	This study
L0 NLS	pAGM1276, oligo NLS F + R	This study
L0 NES	pAGM1276, oligo NES F + R	This study

GoldenGate assembly level 1 (cloning and assembling parts of a gene to make a transcriptional unit)

Annotation	Modules used	Reference
F1-p35S-5U-TMV:Chl-roGFP2-Orp1:tNos	pICH47732, pICH41373, pICH78133, roGFP2-Orp1, pICH41421	This study
F1-p35S-5U-TMV:Mito-roGFP2-Orp1:tNos	pICH47732, pICH41373, pAGM1482, roGFP2-Orp1, pICH41421	This study
R2-pAct2-5U-TMV:Hygro:tAct2	pICH47811, pICH87644, Hygro, pICH44300	This study
F1-p35S-5U-TMV:NLS-roGFP2-Orp1:tNos	pICH47732, pICH41373, pAGT707, pAGM1276, NLS, roGFP2-Orp1, pICH41421	This study
F1-p35S-5U-TMV:NES-roGFP2-Orp1:tNos	pICH47732, pICH41373, pAGT707, pAGM1276, NES, roGFP2-Orp1, pICH41421	This study
F1-p35S-5U-TMV:SP-OsRAmy3A-roGFP2-Orp1:tNos	pICH47732, pICH41373, pICH78141, roGFP2-Orp1, pICH41421	This study
F1-p35S-5U-TMV:roGFP2-Orp1-SKL:tNos	pICH47732, pICH51266, roGFP2-Orp1-SKL, pICH41421	This study
F3-pNos-5U-TMV:Mito-mCherry:tOcs	pICH47751, pICH42211, pAGM1482, pICSL80007, pICH41432	This study

GoldenGate assembly level 2 (assembling different transcriptional unit/gene into a expression vector for plant)

Annotation	Modules used	Reference
F1-p35S-5U-TMV:Chl-roGFP2-Orp1:tNos-R2-pAct2-5U-TMV:Hygro:tAct2	pAGM4723, pICH41744, F1-p35S-5U-TMV:Chl-roGFP2-Orp1:tNos, R2-pAct2-5U-TMV:Hygro:tAct2	This study
F1-p35S-5U-TMV:Mito-roGFP2-Orp1:tNos-R2-pAct2-5U-TMV:Hygro:tAct2	pAGM4723, pICH41744, F1-p35S-5U-TMV:Mito-roGFP2-Orp1:tNos, R2-pAct2-5U-TMV:Hygro:tAct2	This study
F1-p35S-5U-TMV:NLS-roGFP2-Orp1:tNos-R2-pAct2-5U-TMV:Hygro:tAct2	pAGM4723, pICH41744, F1-p35S-5U-TMV:NLS-roGFP2-Orp1:tNos, R2-pAct2-5U-TMV:Hygro:tAct2	This study
F1-p35S-5U-TMV:NES-roGFP2-Orp1:tNos-R2-pAct2-5U-TMV:Hygro:tAct2	pAGM4723, pICH41744, F1-p35S-5U-TMV:NES-roGFP2-Orp1:tNos, R2-pAct2-5U-TMV:Hygro:tAct2	This study
F1-p35S-5U-TMV:SP-OsRAmy3A-roGFP2-Orp1:tNos-R2-pAct2-5U-TMV:Hygro:tAct2	pAGM4723, pICH41744, F1-p35S-5U-TMV:SP-OsRAmy3A-roGFP2-Orp1:tNos, R2-pAct2-5U-TMV:Hygro:tAct2	This study
F1-p35S-5U-TMV:roGFP2-Orp1-SKL:tNos-R2-pAct2-5U-TMV:Hygro:tAct2- F3-pNos-5U-TMV:Mito-mCherry:tOcs	pAGM4723, pICH41766, F1-p35S-5U-TMV:roGFP2-Orp1-SKL:tNos, R2-pAct2-5U-TMV:Hygro:tAct2, F3-pNos-5U-TMV:Mito-mCherry:tOcs	This study

Ref 1. Engler C, Youles M, Gruetzner R, Ehnert TM, Werner S, Jones JD, Patron NJ, Marillonnet S (2014) A golden gate modular cloning toolbox for plants. ACS Synth Biol 3: 839-843
